# Migration Inhibitory Factor in Conditioned Medium from Human Umbilical Cord Blood-Derived Mesenchymal Stromal Cells Stimulates Hair Growth

**DOI:** 10.3390/cells9061344

**Published:** 2020-05-28

**Authors:** Hyun Ah Oh, Jihye Kwak, Beom Joon Kim, Hye Jin Jin, Won Seok Park, Soo Jin Choi, Wonil Oh, Soyoun Um

**Affiliations:** 1 Biomedical Research Institute, MEDIPOST Co., Ltd., Seongnam 13494, Korea; hyun_ah82@medi-post.co.kr (H.A.O.); jihyek@medi-post.co.kr (J.K.); genny77@medi-post.co.kr (H.J.J.); sjchoi@medi-post.co.kr (S.J.C.); wioh@medi-post.co.kr (W.O.); 2 Department of Dermatology, Chung-Ang University College of Medicine, Seoul 06974, Korea; beomjoon74@gmail.com; 3 Aesthetic Research Team, Amore Pacific Corporation Research and Development Center, Yongin 17074, Korea; wspark@amorepacific.com

**Keywords:** hair growth, conditioned media, VEGF, MIF, human mesenchymal stromal cells, androgenic alopecia

## Abstract

Conventional therapeutic applications of mesenchymal stromal cells (MSCs) focus on cell replacement and differentiation; however, increasing evidence suggests that most of their therapeutic effects are carried out by their various secretions. This study investigated the application of conditioned medium (CM) from human umbilical cord blood-derived MSCs (hUCB-MSCs) to improve hair growth and developed a method to reliably produce this optimized CM. Primed MSC-derived CM (P-CM) with combinations of TGF-β1 and LiCl was optimized by comparing its effects on the cell viability of dermal papilla cells (DPCs). P-CM significantly increased the viability of DPCs compared to CM. The secretion of vascular endothelial growth factor (VEGF) in DPCs was regulated by the macrophage migration inhibitory factor (MIF) in the P-CM secreted by MSCs. These findings suggest that P-CM can improve the efficacy in hair growth via a paracrine mechanism and that MIF in P-CM exerts hair growth-promoting effects via a VEGF-related β-catenin and p-GSK-3β [SER9] signaling pathway. Furthermore, clinical trials have shown that 5% P-CM improved androgenetic alopecia through producing an increased hair density, thickness, and growth rate, suggesting that this topical agent may be a novel and effective treatment option for patients with androgenetic alopecia.

## 1. Introduction

Hair loss, also known as alopecia or baldness, occurs in two forms—localized and diffuse—with an increasing incidence as men and women age. Common types of hair loss from the scalp include patterned hair loss resulting from genetic programming and sex hormone expression; alopecia areata caused by an autoimmune disease; and telogen effluvium caused by other factors, including medication, pregnancy, or stress [1,2]. Patterned hair loss is divided into androgenetic alopecia for men and female pattern hair loss for women. The symptoms of hair loss and thinning start as bitemporal recession at the frontal hairline, followed by diffuse thinning over the vertex of the scalp [2]. The standard treatment for this condition is either hair follicle transplantation or the stimulation of these follicles with topical or oral medication [3,4,5,6]. However, these methods are not proven to treat hair loss and can have problematic side effects and high costs [7,8]. Therefore, there is significant interest in finding a safe and effective treatment to prevent androgenetic alopecia and promote hair regrowth.

A hair follicle is a regenerative systemic organ that undergoes cycles of growth (anagen), regression (catagen), resting (telogen), and shedding (exogen) [1,9]. Hair follicles comprise dermal papillae (DP) derived from the mesenchyme and dermal sheath, surrounded by a variety of stem cells [10,11]. DP, located at the base of the hair follicle, reach their maximum size in an anagen state when the cell number is double that in telogen. When a hair follicle is in an anagen state, DP stay deep in subcutaneous fat. DP move up to the dermis in catagen. The hair growth cycle is a complex process regulated by growth factors that are considered to affect the microenvironment that maintains hair follicle growth. The hair growth cycle involves the rapid remodeling of both epithelial and dermal components. Epithelial–mesenchymal interactions are essential for hair formation and postnatal hair growth [12,13], and growth factors within and outside the hair follicle stimulate the proliferation and differentiation of dermal papilla cells (DPCs) and keratinocytes [14]. Androgenetic alopecia is characterized by small-sized hair follicles and a shortening anagen phase. A recent study showed that a hair follicle went into a new anagen from the last telogen when the quiescent stem cells were stimulated to promote proliferation in response to signals from the DP [10,15,16].

Mesenchymal stromal cells (MSCs) are an attractive option for the treatment and repair of damaged tissues as they secrete trophic factors that exert their therapeutic effects, rather than relying on their ability to differentiate into several cell types, including mesoderm- and ectoderm-lineage tissues. Tissue injury activates immune and inflammatory cells, as well as changes in inflammatory molecules and immune cells in the microenvironment, inducing the differentiation of MSCs [17]. Paracrine signaling is known to be one of the major modalities underlying the therapeutic efficacy of MSC-based interventions. Umbilical cord blood (UCB) is an attractive source of mesenchymal stromal cells because of its abundance and relative ease of collection. Some studies have demonstrated that primitive human umbilical cord blood-derived MSCs (hUCB-MSCs) have biological advantages over bone marrow or adipose tissue, which suggests that hUCB-MSCs are a useful model for clinical applications of cell therapies [18,19]. Furthermore, hUCB-MSCs cultured with specific substances produce growth factors and regulatory factors that have paracrine effects on surrounding cells. These growth factors have also been shown to influence hair growth by promoting angiogenesis, maintaining anagen hair, and activating hair growth [20].

The trophic factors secreted from stem cells include secretome and extracellular vesicles, which are found in the culture medium, and their composition varies with changes in the culture conditions. Media enriched for these substances is referred to as conditioned medium (CM) [21,22]. The use of CM could be more beneficial due to the easier transport and lower costs involved than in the application of MSCs themselves. Therefore, CM derived from stem cells could be a promising resource for the development of an alopecia treatment [23,24]. Taken together, these results indicate that UCB-MSC-derived CM may have a therapeutic effect on hair loss, while several other studies have suggested that CM obtained from adipose tissue-derived stem cells promotes hair growth in vitro and in vivo [23,25].

Many reports have suggested that the production of CM secreted from stem cells under hypoxic culture conditions affects damaged cells by upregulating and downregulating growth factors [21,26]. Downregulated epidermal growth factor (EGF), upregulated vascular endothelial growth factor (VEGF), platelet-derived growth factor (PDGF), and insulin-like growth factor II (IGF-II) in stem cell-derived CM has been reported to aid in hair regeneration [24,27]. In addition, TGF-β1 induces the inhibition of hair follicle cell growth as the hair growth cycle progresses from the anagen phase to the catagen phase [28,29]. DPCs are important components of hair follicles and play a critical role in their development through crosstalk with the microenvironment, including the Wnt signaling pathway and surrounding stem cells. It is important to understand that stem cells interact with DPCs to trigger hair regeneration. Wnt signaling is also known to regulate hair morphogenesis and regeneration. By activating the Wnt/β-catenin pathway in DPCs, elevated hair growth was observed. In a previous study, the inhibition of GSK-3β by valproic acid and lithium chloride (LiCl) mimicked the effect of Wnt signaling and promoted hair regeneration [15,30,31,32]. Previous reports suggest that CM from Wnt1a overexpressing MSCs activates DPCs and promotes regeneration of the hair follicle [23]. However, the underlying mechanism of Wnt signaling and its relationship with hair regeneration and DPC stimulation following MSC-derived CM treatment remains unclear.

The aims of this study were to investigate the therapeutic potential of hUCB-MSC-derived CM in the treatment of alopecia, to develop a method for the reliable production of optimized CM to promote hair growth, and to elucidate the underlying mechanism enabling this enhanced hair growth.

## 2. Materials and Methods

### 2.1. hUCB-MSC Culture and CM Preparation

Neonatal umbilical cord blood collected from the umbilical vein was isolated using a Ficoll-Hypaque solution (*d* = 1.077 g/cm^3^; cat# 17-1440-03, Sigma-Aldrich Co., St. Louis, MO, USA), which separated out the mononuclear cells. Separated mononuclear cells were cultured in alpha-minimum essential medium (α-MEM; cat# 12574-048, Gibco, Carlsbad, CA, USA) supplemented with 10% fetal bovine serum (FBS; cat# 16000-044, Gibco) and 50 μg/mL gentamicin (cat# 15710-072, Gibco). Isolated MSCs were incubated at 37 °C in a humidified atmosphere containing 5% CO_2_ and the culture medium was replaced twice a week, as previously described [33]. The yield of MSC isolation was approximately 50%. In total, four out of eight UCB harvests produced MSC-like cells. The cells were passaged to 60%–70% confluency and then used for experiments or redistributed to new culture plates. All subjects (*n* = 8) gave their informed consent for inclusion before they participated in the study. The study was conducted in accordance with the Declaration of Helsinki, and the protocol was approved by the Institutional Review Board of MEDIPOST Co., Ltd. (MP-2015-06).

To collect the conditioned medium (CM) from the MSC cultures, 10 ng/mL transforming growth factor-beta 1 (TGF-β1; cat# PRD240-01, R&D Systems, Minneapolis, MN, USA) and 5 mM lithium chloride (LiCl, cat# L7206, Sigma-Aldrich Co.) in serum-free α-MEM were added to the MSCs (passage 7, 5000 cells/cm^2^) for 1 day, and the culture medium was then changed to serum-free follicle dermal papilla cell growth medium (DPCM; cat# C-26505, Promocell, Heidelberg, Germany). After 3 days, CM from the MSC cultures was collected and used as primed MSC-derived conditioned medium (P-CM), and CM was collected without pre-treatment with TGF-β1 and LiCl to act as the control (Supplementary Figure S1a).

### 2.2. Culture of Follicle Dermal Papilla Cells

Primary human DPCs (55, female, Caucasian) were purchased from Promocell (cat# C-12071, Heidelberg, Germany), and these cells were isolated from human dermis from the lateral scalp and maintained in Dulbecco’s modified Eagle’s medium (DMEM; cat# SH30243.01, Hyclone, South Logan, UT, USA) supplemented with 10% FBS (Gibco) and 100 μg/mL streptomycin/100 U penicillin (cat# 15140122, Gibco) in a humidified 5% CO_2_ atmosphere at 37 °C. DPCs were treated with P-CM from passage five (Supplementary Figure S1b).

### 2.3. Cell Viability Assay

The cell viability was evaluated by the CCK-8 assay (cat# CK04-01, Dojindo, Rockville, MD, USA). DPCs were plated in 96-well flat-bottom tissue culture plates at a density of 4 × 10^3^ cells/well and incubated for 24 h in DMEM with 10% FBS. DPCs were then cultured for an additional 48 h with the addition of 10%, 25%, 50%, or 100% P-CM, CM, or recombinant human macrophage migration inhibitory factor protein (MIF; cat# 289-MF, R&D Systems) in serum-free DMEM. After incubation, the medium was replaced with the CCK-8 reagents diluted in DMEM, and the plates were incubated in the dark for an additional 1 h at 37 °C. The optical density was measured at 450 nm using a VERSAmax microplate reader (Molecular Devices, San Jose, CA, USA).

### 2.4. Western Blot Analysis

After undergoing the treatment described earlier, DPCs were washed with ice-cold 1× PBS and lysed with RIPA buffer (cat# 89901, Thermo Scientific, Waltham, MA, USA) containing a protease and phosphatase inhibitor cocktail (cat# 1861281, Thermo Scientific). Protein concentrations were determined using a BCA Protein assay (cat# 23225, Thermo Scientific). The lysates were separated using Novex, NuPAGE, and Bolt precast gels (Invitrogen, Carlsbad, CA, USA) under denaturing conditions and transferred to nitrocellulose membranes. After blocking with 5% bovine serum albumin solution for 1 h at room temperature, the membranes were immunoblotted with various antibodies (anti-human phospho-GSK-3β [SER9], cat# 9323; anti-human β-catenin, cat# 9562; anti-human phosphor-AKT [SER473], cat# 9271; anti-human cyclin D1, cat# 2978; and anti-human GAPDH; cat# 5174, Cell Signaling, Danvers, MA, USA) overnight at 4 °C, and then probed with horseradish peroxidase-conjugated secondary antibodies for 1 h at room temperature. The bands were visualized using an enhanced chemiluminescence immunoblotting system (GE Healthcare Life Sciences, Buckinghamshire, UK).

### 2.5. Growth Factor Array

The human growth factor array (cat# AAH-GF-1, RayBiotech, Inc., Noncross, GA, USA) was used to evaluate the growth factors secreted from MSCs or DPCs. The DPCs were plated in 60-mm culture dishes at 2 × 10^5^ cells and incubated for 24 h. They were then cultured for 48 h with 50% P-CM in FBS-free DMEM medium, and the culture supernatants were collected. A cytokine antibody array was conducted according to the manufacturer’s protocol (Supplementary Figure S2). The membranes were incubated in blocking buffer at room temperature (RT), and the membranes were then incubated with 1 mL supernatants collected from P-CM or P-CM-treated DPCs for 4 h at RT. The membrane was washed and then incubated with a biotinylated antibody cocktail. The antibodies bound to the array were detected using HRP-streptavidin and an enhanced chemiluminescence detection system, and the spot intensity was quantified using densitometry on Image J software.

### 2.6. L507 Antibody Array

The RayBio^®^ Label-based L-Series Human Antibody Array L-507 (cat# AAH-BLG-1, RayBiotech) was used to assay over 507 cytokines, chemokines, growth factors, soluble receptors, and other proteins in P-CM. From 3.2 × 10^5^ MSCs (P7), 1 mL CM and P-CM were collected. The CM was removed from the serum using dialysis (buffer: 1× PBS, pH 8.0) overnight at 4 °C. CM was dialyzed into a clean microfuge tube and centrifuged at 10,000 rpm for 5 min to remove any particulates or precipitates. The biotin-labeled CM was diluted two-fold in blocking buffer and then incubated with gentle rocking for 2 h at RT. The glass slides were washed five times in wash buffer at RT, and Cy3-conjugated streptavidin was added and incubated for 2 h at RT. The data from this study was analyzed using the RayBio Analysis Tool software, which is available to users of the RayBio Biotin Label-based Antibody Array (EBIOGEN Inc, Seoul, Korea). Each set of data from P-CM and CM was normalized to raw media to minimalize the factors from media.

### 2.7. ELISA

To determine VEGF secretion levels, DPCs were plated in 60-mm culture dishes at a density of 2 × 10^5^ cells and incubated for 24 h. They were then cultured for an additional 48 h with 25% CM in serum-free DMEM medium, and the culture supernatant was collected and analyzed using a VEGF Quantikine ELISA kit (cat# DVE00, R&D Systems). For the analysis of MIF protein levels, CM and P-CM secreted from four different MSC cultures were collected and tested using an MIF ELISA, which was performed according to the manufacturer’s instructions. MIF secretion levels were measured using a human MIF Quantikine ELISA Kit (cat# DMF00B, R&D Systems).

### 2.8. Neutralizing Antibody

To evaluate the effects of VEGF and MIF in DPCs, 25% PM-CM DMEM was treated with neutralizing MIF antibody (1:400, cat# MAB289, R&D systems) or neutralizing VEGF antibody (100 ng/mL, cat# MAB293, R&D Systems) for 48 h. The DPC expression of β-catenin, p-GSK-3β [SER9], p-AKT, and cyclin D1 was then assayed as before.

### 2.9. Clinical Study

This study was designed to be run as a double-blind placebo-controlled clinical trial conducted on physically and mentally healthy adults (aged between 20 and 60 years) who had been diagnosed with mild-to-moderate patterned hair loss (males: Type II according to the modified Norwood–Hamilton classification, women: Ludwig classification Type I). A total of 30 patients completed the 16-week study (P-CM group = 16, placebo group = 14). During clinical trials, we excluded two participants who wanted to withdraw from the clinical trial. Participants who had a history of any skin or scalp disorders, endocrine abnormalities, or systemic diseases such as liver function abnormality were excluded from this study. For preventing any influence of other types of treatment on our results, we excluded any individual who had previously undergone androgenetic alopecia treatment, including the administration of finasteride, an applied topical hair restorer, or surgical treatment for androgenetic alopecia, such as a hair transplant or scalp reduction. Pregnant and nursing women were also excluded.

The topical agent used in this study comprised 5% hUCB-MSC-derived CM pretreated with LiCl and TGF-β1. The placebo included 5% DPCM and was provided to the control group under the same conditions. Both groups were asked to apply this agent twice daily (morning and evening, 1–2 mL/dose) and then massage the scalp after application for 1–2 min for a total of 16 weeks. The hair density and diameter were objectively assessed using a phototrichogram (Folliscope 4.0; Lead M, Seoul, Republic of Korea) and compared to the patient baseline data (baseline) after 4, 8, and 16 weeks of treatment. The hair density (hair count/cm^2^) was calculated by counting the total number of hairs in the target area. The hair thickness (mm) and hair growth rate (mm/day) were calculated as the average diameter of five hairs measured manually in the target area. The formula for determining the rate of hair growth was as follows: Hair growth rate = (Hair length 3 days after shaving − Hair length immediately after shaving) / passed days. Prior to conducting hair measurements, patients who had hair in the same area affected by patterned hair loss were asked to shave a circle with a diameter of 1 cm near the crown of the head to a length of <2 mm. The target area at the center of the circle was marked with a 1-mm black dot (tattoo).

To determine the hair density in the 24-week study, 5% P-CM treatment was run separately as a double-blind placebo-controlled clinical trial with 52 patients. During clinical trials, we excluded nine participants who dyed their hair or applied other hair restorer. Overall, 43 participants (CM group = 25, placebo group = 18) completed the clinical trial. After 24 weeks of treatment, the hair density (each/cm^2^) was measured and a professional visual assessment was performed using a phototrichogram (Folliscope 4.0), and the results were compared to the baseline data. The hair density (each/cm^2^) was calculated by counting the total number of hairs in the target area. For the professional visual assessment, the score was determined by comparing the results with the patient baseline data (baseline) after 8, 16, and 24 weeks of treatment: +3, excellent; +2, good; +1, moderate; 0, no change; −1, poor; −2, very poor; −3, bad [34]. This study was approved by the KC Skin Research Center. The participants in the clinical trial provided written informed consent prior to study participation (KC-IRB-018).

### 2.10. Flow Cytometry

MSCs isolated from cord blood were labeled with the following antibodies: FITC-conjugated human CD14 (cat# 555397), CD45 (cat# 555483), HLA-DR (cat# 555811), PE-conjugated human CD73 (cat# 550257), CD166 (cat# 559263, BD Biosciences), CD90 (cat# 12-0909-42), and CD105 (cat# 12-1057-42, Invitrogen). Isotype controls were also included: PE-conjugated Isotype Control (cat# 555743) and FITC-conjugated Isotype control (cat# 555573, BD Biosciences). Stained cells were analyzed by flow cytometry on a MACSQuant instrument (Miltenvi Biotec, Bergisch Gladbach, Germany).

### 2.11. Statistical Analysis

All data are presented as the mean ± standard deviation (SD), and each of the values were calculated using experimental data repeated at least three times. Statistical analysis was performed using a one-way nonparametric and two-tailed *t*-test analysis of variance (ANOVA), followed by a Fisher’s least significant difference (LSD) test on Prism 7 software (GraphPad, San Diego, CA, USA). A statistically significant difference was reported if *p* < 0.05.

In the clinical study, statistical analyses were carried out using SPSS (v23.0; IBM SPSS, Armonk, NY, USA) software. These analyses were used to assess the efficacy of the test product. Statistically significant differences between week 0 and 16 of the same group were determined by paired-simple *t*-tests. Statistically significant differences between the test and control groups were determined by the Mann–Whitney U test. For parametric tests, the level of significance was set at *p* < 0.05. Statistically significant differences between before and after the use of the product for each group were determined by ANOVA.

## 3. Results

### 3.1. Primed CM Promoted the Cell Viability of DPCs

**Figure 1:**
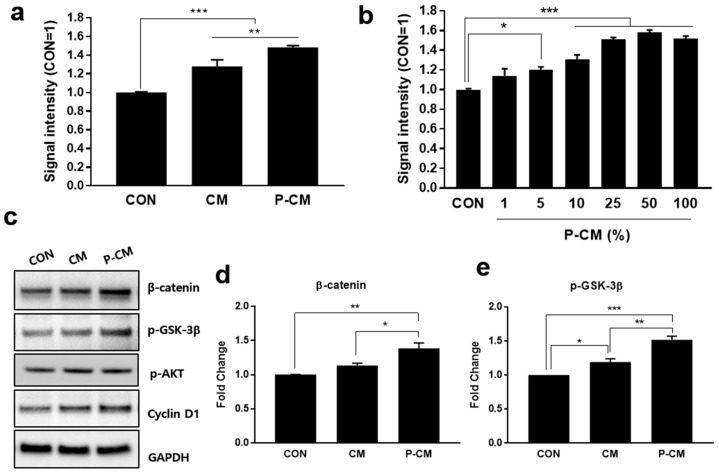
Primed CM (P-CM) induced the viability of DPCs and increased the expression of β-catenin and p-GSK-3β [SER9]. DPCs were treated with 25% CM and P-CM in DMEM for 48 h. DPCs were cultured in DMEM medium without supplementation as a control. (**a**) The cell viability of the DPCs, which was evaluated by the CCK-8 assay, was significantly increased after treatment with 5% P-CM. (**b**) The data shows that the viability of DPCs increased with an increasing P-CM concentration. (**c**–**e**) Western blot analysis: After treating DPCs with 25% CM or P-CM for 48 h, cell lysates were collected and subjected to western blot analysis with specific antibodies. β-catenin and p-GSK-3β [SER9] expression in P-CM-treated DPCs was significantly increased. In addition, there was also an increase in p-AKT and cyclin D1 in P-CM-treated cells. The data are reported as the fold change in comparison to the control group (CON). Data represent the mean ± SD. Each experiment was repeated at least three times. Statistically significant differences were determined by one-way nonparametric ANOVA. * *p* < 0.05, ** *p* < 0.01, and *** *p* < 0.001. Abbreviations: CON, raw Dulbecco’s modified Eagle’s medium (DMEM); CM, mesenchymal stromal cell (MSC)-derived conditioned medium; P-CM, primed MSC-derived conditioned medium; DPCs, follicle dermal papilla cells.

To investigate the effect of primed conditioned medium (P-CM) on DPCs, MSCs derived from human UCB were first treated with TGF-β1 and LiCl for 1 day. After priming, the medium was changed and incubated with DPCM for 3 days, after which P-CM was collected (Supplementary Figure S1). CM and P-CM were used to treat DPCs for 48 h and these cultures were then subject to the CCK-8 assay to determine the cell viability, although the important consideration, changes in the intracellular metabolic activity, still remained. The cell viability was improved in both 25% CM and 25% P-CM in DMEM when compared to the untreated medium. P-CM (with TGF-β1 and LiCl) improved the DPC cell viability when compared with unprimed CM (Figure 1a and Supplementary Figure S6). The DPC viability was seen to increase in a P-CM concentration-dependent manner (Figure 1b and Supplementary Figure S6). This data suggests that CM and P-CM had some positive effect on the cell viability, and that this effect was enhanced in CM produced from cells pre-treated with TGF-β1 and LiCl.

It is well-known that activating the Wnt/β-catenin signaling pathway by inhibiting GSK-3β induces β-catenin phosphorylation and degradation, promoting DPC proliferation [15,20,23,30]. Therefore, we examined the protein levels of β-catenin, phosphorylated GSK-3β (p-GSK-3β [SER9]), p-AKT, and cyclin D1, which are all known to mediate cell proliferation, by western blot analysis. P-CM treatment was shown to increase β-catenin, p-GSK-3β [SER9], phosphorylated AKT, and cyclin D1 when compared to the control and CM-treated groups. Our results suggested that CM and P-CM significantly enhance the DPC viability while activating β-catenin via the inhibitory [SER9] phosphorylation of GSK-3β (Figure 1c–e; Supplementary Figure S3). Taken together, these findings imply that the proteins, cytokines, cell-signaling molecules, and growth factors secreted by MSCs primed with TGF-β1 and LiCl might promote the viability of DPCs via the β-catenin signaling pathway.

### 3.2. P-CM Enhanced the Secretion of Growth Factors in DPCs

**Figure 2:**
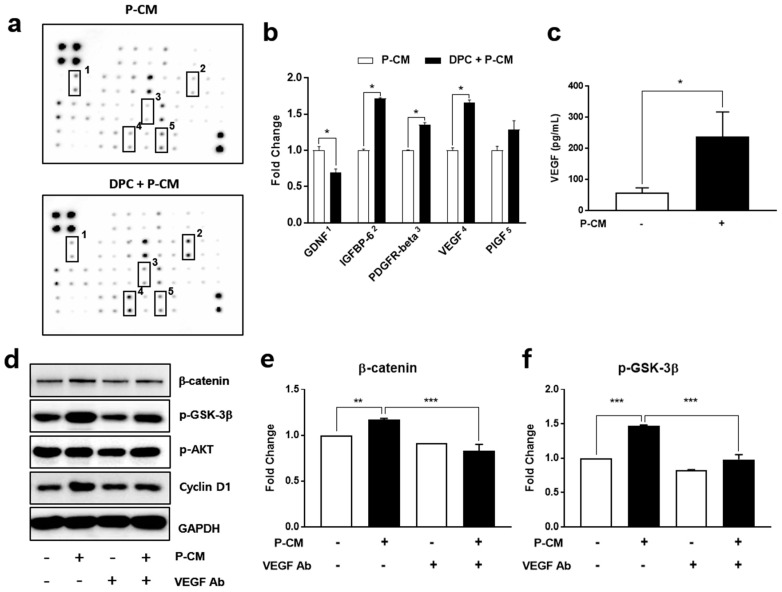
Secreted protein array from DPCs treated with P-CM. (**a**) Human growth factors were identified using an antibody array following DPC treatment with 50% P-CM for 48 h. (**b**) The levels of five growth factors were significantly changed when compared to 50% P-CM only in DMEM. This increased secretion was quantified and analyzed using optical intensity analysis. The growth factor array demonstrated that P-CM significantly increased VEGF secretion in DPCs. Four proteins, consisting of IGFBP-6, PDGFR-beta, VEGF, and PIGF, were also elevated. Data was evaluated for the fold increase, normalized to the intensity of these proteins in P-CM only, which was defined as 1. On the other hand, the level of GDNF was significantly downregulated in DPCs. (**c**) To confirm the upregulation of VEGF in DPCs following P-CM treatment, the VEGF secretion levels were measured by ELISA. (**d**–**f**) Neutralizing VEGF antibody was used to block the increased VEGF in DPCs to confirm the hypothetical role of this protein. When VEGF was neutralized, there was a corresponding reduction in β-catenin, p-GSK-3β [SER9], p-AKT, and cyclin D1 in DPCs, confirming that VEGF is likely to be a key regulator in this cascade. Data represent the mean ± SD. Each experiment was repeated at least three times, except for the protein array. The protein array was repeated two times. Statistically significant differences were determined by a two-tailed and unpaired *t*-test. * *p* < 0.05, ** *p* < 0.01, and *** *p* < 0.001. Abbreviations: P-CM, primed MSC-derived conditioned medium; DPCs, follicle dermal papilla cells; VEGF, vascular endothelial growth factor; IGFBP-6, insulin-like growth factor-binding protein; PDGFR, platelet-derived growth factor receptor; PIGF, placental growth factor; GDNF, glial cell-derived neurotrophic factor.

To determine which factors in the P-CM influence the DPC viability, we identified the growth factors secreted by DPCs after treatment with P-CM using a growth factor array. A screening of growth factor production was performed to discover the mechanisms underlying hair growth by P-CM secreted from MSCs. When we compared the results for P-CM-treated DPC secretion and P-CM, P-CM induced a greater secretion of glial cell-derived neurotrophic factor (GDNF), insulin-like growth factor-binding protein (IGFBP-6), platelet-derived growth factor receptor (PDGFR)-beta, placental growth factor (PlGF), and VEGF in DPCs. There was a significant (0.7-fold) decrease in GDNF in DPCs following P-CM treatment, while PlGF (1.3-fold), IGFBP-6 (1.71-fold), PDGFR-beta (1.36-fold), PlGF (1.3-fold), and VEGF (1.66-fold) were all increased following P-CM treatment (Figure 2a,b and Supplementary Table S1).

We confirmed our observations using a VEGF ELISA on DPCs treated with P-CM. In the P-CM treatment group, we observed a three-fold increase in the VEGF protein levels (Figure 2c). Western blot analysis showed that the expression levels of β-catenin and phosphorylated GSK-3β [SER9] increased in DPCs treated with P-CM. Using a neutralizing antibody against VEGF, we were able to reverse the P-CM-induced changes to β-catenin and p-GSK-3β [SER9], demonstrating that the regulation of these proteins was predominantly due to the activity of VEGF (Figure 2d). Taken together, these results demonstrate that P-CM enhances the secretion of VEGF in DPCs, triggering an improved growth of DPCs via the β-catenin and p-GSK-3β [SER9] signaling pathway.

### 3.3. MIF Expression was Increased in MSCs with Priming

**Figure 3:**
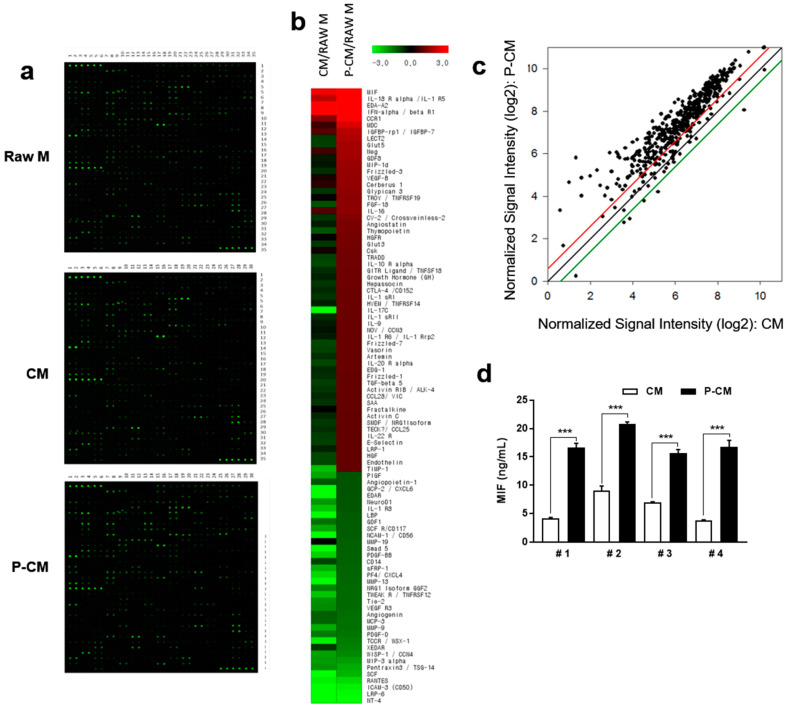
Priming MSCs with TGF-β1 and LiCl resulted in a significantly increased secretion of MIF. (**a**,**b**) CM and P-CM, collected from supernatants of MSCs after priming with or without TGF-β1 and LiCl, were subjected to an antibody array, and the dot intensity was then analyzed. The normalized log2 expression of cytokines, chemokines, and proteins in P-CM showed significant differences (*p* < 0.05) between the CM and P-CM groups, which is represented by the color scale. MIF levels in MSCs with priming were significantly increased. (**c**) After being normalized to Raw M, the normalized intensities of CM and P-CM were the logarithm to base 2 for comparison. Detailed increases (1.5-fold, red line) and decreases (1.5-fold, green line) of P-CM compared to CM are shown. (**d**) The secretion of MIF was measured by ELISA in four lots of UCB-MSCs after priming with TGF-β1 and LiCl. Data represent the mean ± SD. ELISA was repeated four times in triplicate. The growth factor array was repeated two times. Statistically significant differences were determined by one-way nonparametric ANOVA. *** *p* < 0.001. Abbreviations: MSC, mesenchymal stromal cells; MIF, macrophage migration inhibitory factor; CM, MSC-derived conditioned medium; P-CM, primed MSC-derived conditioned medium; Raw M, raw medium; UCB, umbilical cord blood.

MSC-derived CM contains various growth factors, cytokines, and proteins secreted by the stromal cells, which function in tissue regeneration. To identify potential targets related to the increased cellular proliferation on DPCs following MSC priming with TGF-β1 and LiCl, growth factor expression profiling of P-CM was performed using 507 antibodies and compared to CM (Supplementary Table S2). CM and P-CM were normalized to raw medium. The results show that 57 growth factors were significantly upregulated and 35 were significantly downregulated in P-CM normalized to raw medium. The ratio of P-CM to CM is shown in Table 1. MIF was upregulated four-fold in P-CM compared to CM (Figure 3a–c and Table 1). MIF was significantly upregulated in MSCs pretreated with TGF-β1 and LiCl. Four different lots of MSCs were tested to evaluate variations in priming. The secretion of MIF was found to have increased by approximately three-fold compared to that in CM without significant variation (Figure 3d). Additionally, there was no change in surface markers related to stem cell characteristics. This indicates that MSCs did not lose their stemness with priming (Supplementary Figure S4).

### 3.4. MIF-Regulated P-CM Induced VEGF Secretion

**Figure 4:**
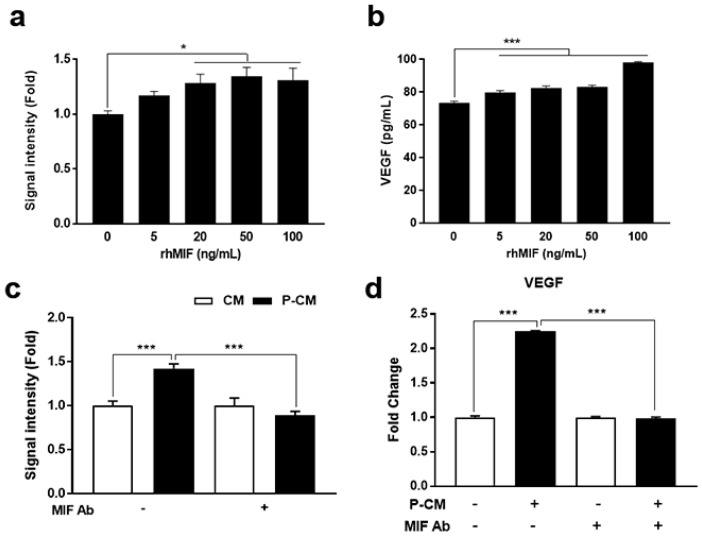
Increased levels of MIF in MSCs, following priming, increased the cell viability by regulating the secretion of VEGF in DPCs. (**a**) The cell viability of DPCs, measured by CCK-8, demonstrated an increased viability in the presence of recombinant human MIF proteins. (**b**) The supernatants of DPCs were collected to analyze the secretion of VEGF after treatment with varying concentrations of MIF proteins. (**c**) To confirm the effect of MIF on the viability of DPCs, MIF-neutralizing antibodies were added with CM or P-CM. The cell viability of DPCs was reduced following MIF neutralizing antibody exposure. (**d**) The increased VEGF levels following the P-CM treatment of DPCs was significantly impaired following the addition of the MIF neutralizing antibodies. Data represent the mean ± SD. Each experiment was repeated at least three times. Statistically significant differences were determined by one-way nonparametric ANOVA. * *p* < 0.05 and *** *p* < 0.001. Abbreviations: MIF, macrophage migration inhibitory factor; CM, MSC-derived conditioned medium; P-CM, primed MSC-derived conditioned medium; VEGF, vascular endothelial growth factor; MIF, macrophage migration inhibitory factor.

We examined whether MIF regulated DPC viability and VEGF secretion. DPCs were treated with various concentrations of recombinant MIF proteins for 48 h. The cell viability of the DPCs significantly increased in a concentration-dependent manner with increasing MIF concentrations (Figure 4a). MIF treatment significantly stimulated VEGF secretion in a dose-dependent manner (Figure 4b). Using a neutralizing antibody for MIF, the elevated cell viability and VEGF secretion in P-CM-treated DPCs was suppressed (Figure 4c,d). These results suggest that MIF is a key regulator of the increased DPC viability for recruitment of the DPC conduction ability induced by P-CM treatment. MIF secreted by MSCs primed with TGF-β1 and LiCl regulates VEGF secretion in DPCs, enhancing their viability.

### 3.5. Topical Agents Containing P-CM Improved Androgenetic Alopecia

Toxicity studies were conducted with P-CM pursuant to the MFDS guidelines for cosmetic products to confirm that the conditioned medium is safe for use in topical application (Supplementary Data S1). The mean patient (*n* = 30) age was 46.9 years (range: 33–55 years). The mean ages of the P-CM and placebo groups were 46.0 and 47.9, respectively, which were not significantly different (*p* > 0.05). Males comprised 3.33% of the study population (1/30 individuals) and females comprised 96.67% (29/30). None of the patients included in the study used any products or were taking any drugs that could be reasonably expected to influence hair growth for at least 6 months prior to the start of this study.

**Figure 5:**
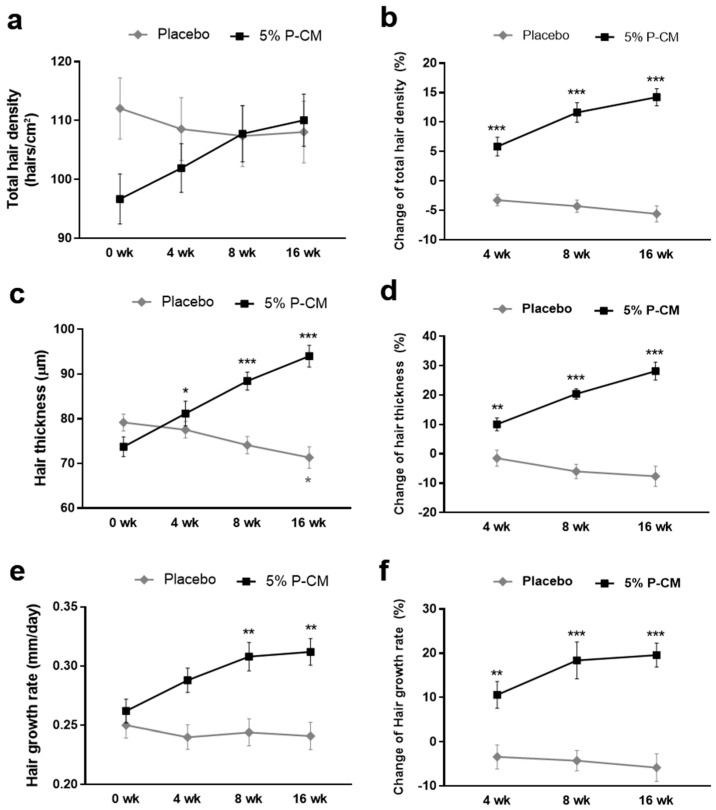
Clinical study of hair growth in participants using P-CM at 4, 8, and 16 weeks. The results of the clinical study suggest that a hair tonic containing 5% P-CM could help to treat androgenetic alopecia. Measurements of the (**a**,**b**) hair density, (**c**,**d**) hair thickness, and (**e**,**f**) hair growth rate showed a significant increase in the 5% P-CM-treated group compared to the placebo group. Statistically significant differences for the (**a**) total hair density, (**c**) hair thickness, and (**e**) hair growth rate were determined between week 0 and 16 of the same group. For the change of the (**b**) hair density, (**d**) hair thickness, and (**f**) hair growth factor, a significant difference of P-CM was determined compared to the placebo group. * *p* < 0.05, ** *p* < 0.01, and *** *p* < 0.001. Abbreviations: P-CM, primed MSC-derived conditioned medium.

The mean hair density in the P-CM group increased from 96.69 ± 16.989 to 110.06 ± 17.726 hairs/cm^2^ over the 16-week treatment period (*p* < 0.001), which represented an increase of 14.24%. Conversely, the hair density of the placebo group decreased from a mean of 114.64 ± 20.78 hairs/cm^2^ prior to treatment to 108.07 ± 19.59 hairs/cm^2^ after 16 weeks. The increase in hair density experienced by the P-CM group was statistically significant when compared to the values of the placebo group (*p* < 0.001) (Figure 5a,b). To demonstrate the long-term effect of P-CM, the hair density in the two groups was evaluated over a 24-week treatment period. Overall, the P-CM groups showed a significant increase in hair density (Supplementary Figure S5).

The mean hair thickness in the test group increased from 0.074 ± 0.009 mm to 0.094 ± 0.010 mm (*p* < 0.001), representing an increase of 28.19%. Conversely, the control group had a hair thickness of 0.078 ± 0.007 mm prior to treatment, 0.076 ± 0.007 mm after 4 weeks, 0.073 ± 0.007 mm after 8 weeks, and 0.071 ± 0.009 mm after 16 weeks. The differences in hair thickness between the groups were not statistically significant (*p* > 0.05); however, at 16 weeks, the test group showed a statistically significant increase in hair thickness compared with the control group (*p* < 0.001) (Figure 5c,d).

The change in the rate of hair growth during the study in the treatment and control groups was analyzed by evaluating changes in hair length over 16 weeks. The mean rate of hair growth in the test group increased from 0.262 ± 0.039 to 0.312 ± 0.045 mm/day over the 16-week study period (*p* < 0.001), which was an increase of 19.54%. In contrast, the rate of hair growth decreased from a mean of 0.257 ± 0.039 mm/day prior to treatment to 0.241 ± 0.043 mm/day after 16 weeks (*p* > 0.05). The increase in the rate of hair growth noted for the test group was statistically significant compared with that of the control group (*p* < 0.001) (Figure 5e,f).

Taken together, the results of this study indicate that the application of a topical agent (cosmetic formulation) containing P-CM at 5% (v/v), as used in this study, can induce statistically significant improvements in the hair density, hair thickness, and hair growth rate, when compared with those in a placebo group. No severe adverse effects were noted for any of the patients in this study. Likewise, none of the patients reported irritation or itching.

## 4. Discussion

**Figure 6:**
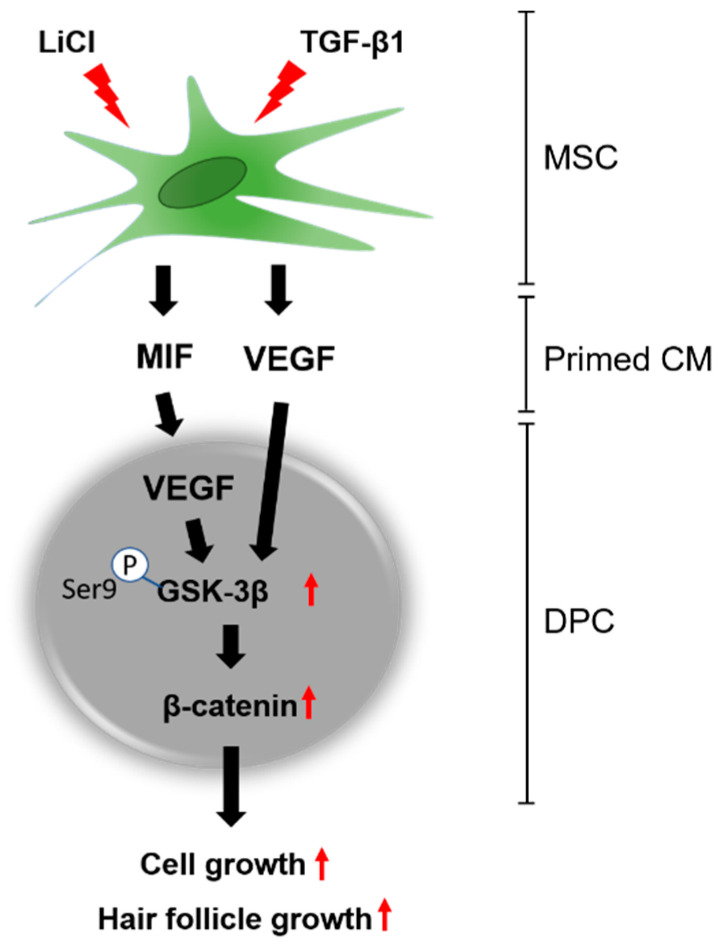
Schematic diagram of DPC proliferation and hair follicle growth. Increased MIF in P-CM secreted by MSCs was induced by TGF-β1 and LiCl. MIF in P-CM activates VEGF secretion in the DPCs, followed by the activation of the inhibitory phosphorylation GSK-3β and phosphorylation of β-catenin, which increases the ubiquitination and thereby the degradation of β-catenin. Overall, P-CM regulated the viability of DPCs and hair follicle growth via the activation of VEGF in DPCs. Abbreviations: MSCs, mesenchymal stromal cells; MIF, macrophage migration inhibitory factor; P-CM, primed MSC-derived conditioned medium; DPCs, follicle dermal papilla cells; VEGF, vascular endothelial growth factor.

MSCs are living cells that can adapt to disease environments by changing their pattern of trophic factor secretion. MSC-derived CM is a promising pharmaceutical agent for regenerative medicine, including treatment for hair loss. CM is a collection of trophic factors free from MSCs; thus, it should be possible to tailor this CM by pretreating MSCs in order to induce the secretion of specific factors optimized for the promotion of hair growth [21,22,23]. For pretreatment, we first exposed MSCs in vitro to an artificial environment mimicking an alopecia state in hair follicles (“priming”). Human hair follicles affected by androgenic alopecia showed dramatic decreases in viability and proliferation. Dermal papilla cells (DPCs) on hair follicles manage hair follicle cycling by inducing signaling factors related to cell growth. The restoration of hair induction by DPC stimulation is considered a potential therapy for hair loss. In this study, we demonstrated that conditioned medium (CM), collected from MSCs primed with TGF-β1 and LiCl, upregulated the viability of DPCs and hair growth. MIF, secreted by primed MSCs, induced VEGF in the DPCs by inhibiting GSK-3β, which induces β-catenin phosphorylation (Figure 6).

The paracrine effect is known to play a key role in the therapeutic efficacy of MSCs. When MSCs are administered in the body, they adapt to the new environment in the damaged areas and start to secrete various trophic factors, including cytokines and growth factors, which play roles in the anti-inflammatory response, the induction of anti-apoptotic factors, mitogenesis, and the activation of endogenous stem cells [35,36,37]. The therapeutic effects of the trophic factors secreted by MSCs are collectively referred to as the paracrine effect or paracrine action of MSCs. CM, containing growth factors and tissue regenerative agents secreted by MSCs, has a paracrine effect on neighboring cells [38]. Previous research has shown that cytokines secreted by umbilical cord blood-derived and adipose-derived stem cells promote hair growth [20,22,23,24]. Additionally, one clinical study demonstrated that the hair stimulating complex produced by skin cells grown under hypoxic conditions secreted high levels of Wnt protein and had an efficacy in hair restoration [39]. Therefore, conditioned medium secreted by primed cells had the possibility to regulate hair growth. In this study, we treated MSCs with TGF-β1 and LiCl to induce the secretion of cytokines involved in the stimulation of hair growth by mimicking the conditions of alopecia.

In hair follicles, the high expression of TGF-β induces growth suppression and cell apoptosis in the hair follicular matrix and epithelial strands through a shift in hair cycle progression from the anagen to the catagen phase [28,29]. It has become apparent that TGF-β is deeply involved in the progression of alopecia. TGF-β1 knockout mice have hair follicles that exhibit anagen phase elongation, and a shift to the catagen phase can be induced by TGF-β1 administration. These findings suggest that TGF-β1 functions as a regression phase inducer of hair follicles and that controlling TGF-β signaling may be an effective strategy for alopecia treatment. TGF-β has been shown to induce a catagen-like morphology and inhibit human hair follicle growth in vitro. Previous studies have reported that the production of TGF-β1 and hair growth inhibitory factors from DPCs is enhanced by androgens [40,41,42]. It is interesting that the TGF-β family mimetic peptide promotes the proliferation of human follicle dermal papilla cells and hair growth [43]. Lithium chloride (LiCl) is an agonist of canonical Wnt signaling that inhibits GSK-3β activity and thereby effectively stabilizes free cytosolic β-catenin [44,45]. Specifically, one report showed that TGF-β2 plays a prominent role in dampening BMP signaling and facilitates the activation of hair follicle stem cells [46]. Another study showed that the inhibition of TGF-β signaling in hair follicles during telogen to early anagen leads to the blockade of anagen re-entry [47]. Taken together, these results strongly indicate that TGF-β signaling plays an important role in regulating the proliferation, differentiation, and apoptosis of distinct stem cell populations in hair follicles. Furthermore, these findings indicate the therapeutic potential of hUCB-MSC-derived CM pretreated with LiCl and TGF-β in androgenetic alopecia. Therefore, TGF-β and LiCl appear to contribute to maximizing the efficacy of CM. We found that conditioned medium (P-CM) obtained by priming with a combination of TGF-β1 and LiCl resulted in a significantly enhanced viability of DPCs. However, these two potent agents might have masked the efficacy of CM alone. To clarify this, a further in vivo study is required.

Hair follicle regeneration begins when signals from the DPCs reach multipotent epidermal stem cells in the bulge region. Androgenetic alopecia involves the action of dihydrotestosterone (DHT) on the DPCs that line the base of the hair follicle. However, the mechanism behind this action is not completely understood. Circulating androgens act on DPCs and alter paracrine factors that influence hair epithelial cells. According to a previous study, DHT shortens the duration of the hair growth cycle by initiating cell-cycle arrest and downregulating levels of β-catenin. Specifically, DHT decreases the levels of total and nuclear β-catenin, an important regulator of hair growth and proliferation, while LiCl, a glycogen synthase kinase-3β inhibitor, attenuates DHT-induced downregulation of β-catenin [48]. LiCl consistently reverses the effect of DHT on the ability of dermal papilla cells to induce hair follicle stem cell differentiation. For example, one study showed that conditioned medium from DPCs treated with LiCl and DHT induced the expression of K6hf, indicating that the activation of Wnt ⁄β-catenin by LiCl in DHT-treated DPCs rescues the capacity for hair cell differentiation [49]. Taken together, these results suggest that at least some of the paracrine factors involved in hair differentiation are target genes in the Wnt/β-catenin signaling pathway.

Hair follicle regeneration begins when signals from mesenchyme-derived DPCs reach multipotent epidermal stem cells in the bulge region of the follicle [11]. Several signaling molecules involved in the Wnt, BMP, shh, and TGF-β signaling cascades have been shown to be involved in the normal hair follicle cycle [10,14]. The Wnt/β-catenin pathway is one of the most important elements in hair growth regulation [15]. β-Catenin is markedly activated in DPCs during the anagen phase and is important for regeneration of the hair cycle. The inhibition of glycogen synthase kinase 3 (GSK-3) results in the hypo phosphorylation of β-catenin, as well as its increased abundance and nuclear accumulation; this promotes the transcription of growth-promoting gene products cyclin D1 and p21, which are responsible for the cell cycle [15,50,51]. Wnt/β-catenin signaling activation by inhibiting GSK-3β maintains the ability of human DPCs to induce hair growth, and β-catenin activity via p-Akt in the DP regulates morphogenesis and the regeneration of hair. In our study, we observed increases in p-GSK-3β [SER9] and β-catenin expression following DPC treatment with P-CM, and demonstrated that the effect of β-catenin on hair growth can be attributed to its influence on DPCs. Although cyclin D1 and p-AKT were not significantly changed, the P-CM treatment of DPCs increased the expression of cyclin D1 and p-AKT, which are both related to the proliferation mechanism of DPCs.

To investigate the hair growth-promoting effects of the MSC secretions, the cytokine expression profile of these cells was verified using an antibody array. The results revealed that GDNF, IGFBP-6, PDGFR-beta, PlGF, and VEGF all increased after the P-CM-treatment of DPCs. There is evidence that IGF and IGFBP exert a positive effect on the cell viability and VEGF secretion via β-catenin signaling in DPC [20,24]. In addition, PlGF and VEGF expression is known to mediate hair growth [7,24,52]. In this study, primed CM significantly induced the secretion of VEGF in DPCs. VEGF is a strong regulator of pathological angiogenesis and regulates DPC proliferation [53,54,55]. VEGFR2 is a well-known VEGF receptor, and β-catenin and AKT are downstream proteins that are regulated by VEGF [56,57,58]. When we treated cells with P-CM, β-catenin and p-GSK-3β [SER9] expression in DPCs was significantly increased. When cells were treated with a neutralizing VEGF antibody, the increased expression of β-catenin and p-GSK-3β [SER9] was suppressed. Overall, these results suggest that VEGF is a key mediator of DPCs and promotes cell viability via β-catenin/GSK-3β signaling, triggered by treatment with TGF-β1/LiCl-primed MSCs.

These findings suggested that a further examination of the specific factors in the P-CM collected was warranted. We hypothesized that a specific factor in CM could be responsible for the effects of primed CM. To examine this possibility, the growth factors secreted by the MSCs primed with TGF-β and LiCl were analyzed using an L507 growth factor array. This analysis revealed at least 88 growth factors that were differentially expressed between the control and P-CM. Among these, MIF was shown to be upregulated four-fold in P-CM. MSCs from four different donors showed the continued and elevated secretion of MIF upon priming with TGF-β and LiCl.

MIF was originally identified because of its ability to inhibit the random migration of macrophages in vitro. MIF is expressed in hair follicles, and there is a close association between reduced MIF in hair follicles and the development of alopecia areata, which is an autoimmune disease of the skin [59,60]. Interestingly, treatment with TGF-β1 and LiCl induced the expression of MIF in MSCs, and treatment with MIF induced DPC viability for recruitment of the conduction ability. To obtain a better insight into the relationship between MSC-secreted MIF and VEGF in DPCs, we confirmed that the VEGF expression in DPCs was dependent on MIF. Significant increases in VEGF expression were observed when MIF was introduced, and these changes appeared to be dose-dependent. The increased levels of VEGF in DPCs treated with P-CM were significantly suppressed when a neutralizing MIF antibody was introduced. However, the relationship between the inflammatory, chemotactic, and proliferative capacities of MIF and cancer severity is still unclear. Overexpressed MIF levels in multiple models of cancer have been reported [61]. Therefore, the direct use of recombinant MIF proteins for the treatment of hair loss requires more studies.

Additionally, a double-blind and placebo-controlled clinical study on patients with patterned hair loss demonstrated that the hair density and thickness were significantly increased, while the hair growth rate increased from 0.262 to 0.312 mm/day, with no severe adverse reactions on hair and skin when patients used a topical treatment enriched with 5% v/v P-CM. The improvements caused by 5% v/v P-CM treatment were significantly greater that those observed in placebo-treated sites in terms of the hair density at 4, 8, and 16 weeks. Although the patients were randomized before the treatment, the total density at week 0 showed a dramatic difference between P-CM and placebo groups. This could be due to the difference in the initial disease severity of patients between the two groups. This study lays the groundwork for future work, which will require a larger sample size to confirm the inherent limitation of this clinical data. Notwithstanding the inherent limitation, the changes of the total hair density, hair thickness, and hair growth rate of the P-CM-treated group in the terminal week (16 weeks) showed significant increases compared to the placebo group.

These findings demonstrate that MIF is required for the effects of P-CM and is a key modulator of hair growth-related protein, VEGF, in DPCs. This interaction is mediated via the β-catenin and p-GSK-3β [SER9] signaling pathways. These results strongly suggest that P-CM can counteract hair loss and could be a promising therapeutic agent for hair restoration. In addition, the results of the pilot clinical trial suggest that P-CM derived from MSCs is a safe and effective treatment for androgenetic alopecia. Emerging and future combined treatments with MSCs may lead to a new paradigm in the management of androgenetic alopecia.

## Figures and Tables

**Table 1:** Upregulated and downregulated proteins in CM and P-CM.

Upregulated in P-CM Normalized to Raw Medium				Downregulated in P-CM Normalized to Raw Medium			
Protein	CM	P-CM	P-CM/CM Ratio	Protein	CM	P-CM	P-CM/CM Ratio
	Normalized to Raw Medium				Normalized to Raw Medium		
MIF	5.50	22.62	4.11	PlGF	0.26	0.50	1.96
IL-18 R alpha/IL-1 R5	3.87	8.90	2.30	Angiopoietin-1	0.49	0.50	1.02
EDA-A2	9.72	8.10	0.83	GCP-2/CXCL6	0.07	0.50	6.67
IFN-alpha/beta R1	14.50	7.66	0.53	EDAR	0.04	0.50	12.50
CCR1	2.64	5.97	2.26	NeuroD1	0.29	0.49	1.72
MDC	1.56	4.65	2.99	IL-1 R8	0.20	0.49	2.49
IGFBP-rp1/IGFBP-7	1.90	3.58	1.89	LBP	0.05	0.48	10.35
LECT2	0.60	3.46	5.77	GDF1	0.40	0.47	1.18
Glut5	0.59	3.18	5.36	SCF R/CD117	0.26	0.47	1.83
GDF8	0.78	3.11	1.90	NCAM-1/CD56	0.10	0.47	4.49
MIP-1d	0.80	2.95	3.97	MMP-19	1.06	0.46	0.44
Frizzled-3	0.66	2.88	3.67	Smad 5	0.06	0.46	7.72
VEGF-B	1.28	2.82	4.34	PDGF-BB	0.18	0.45	2.52
Cerberus 1	1.44	2.79	2.21	CD14	0.57	0.45	0.79
Glypican 3	0.64	2.75	1.94	sFRP-1	0.24	0.44	1.85
TROY/TNFRSF19	1.10	2.71	4.29	PF4/CXCL4	0.17	0.44	2.63
FGF-18	0.51	2.69	2.45	MMP-13	0.02	0.43	25.54
IL-16	1.83	2.69	5.27	NRG1	0.34	0.42	1.23
Crossveinless-2	0.64	2.50	1.47	TWEAK R	0.20	0.42	2.10
Angiostatin	0.76	2.37	3.91	Tie-2	0.31	0.42	1.36
Thymopoietin	0.50	2.32	3.12	VEGF R3	0.32	0.42	1.32
HGFR	1.05	2.29	4.65	Angiogenin	0.46	0.41	0.90
Glut3	0.62	2.28	2.19	MCP-3	0.45	0.40	0.90
Csk	1.21	2.21	3.69	MMP-9	0.25	0.40	1.59
TRADD	0.58	2.21	1.82	PDGF-D	0.37	0.38	1.01
IL-10 R alpha	0.55	2.21	3.80	TCCR/WSX-1	0.14	0.36	2.51
GITR Ligand	0.68	2.19	4.01	XEDAR	0.62	0.35	0.56
Growth Hormone	0.75	2.18	3.22	WISP-1/CCN4	0.27	0.33	1.21
Hepassocin	0.63	2.17	2.88	MIP-3 alpha	0.28	0.28	1.01
CTLA-4/CD152	0.69	2.15	3.45	Pentraxin3	0.30	0.25	0.83
IL-1 sRI	0.64	2.15	3.10	SCF	0.03	0.23	8.86
HVEM/TNFRSF14	0.74	2.15	3.35	RANTES	0.14	0.17	1.18
IL-17C	0.10	2.14	2.92	ICAM-3 (CD50)	0.12	0.14	1.18
IL-1 sRII	0.68	2.14	22.31	LRP-6	0.02	0.13	8.22
IL-9	0.76	2.13	3.16	NT-4	0.10	0.11	1.18
NOV/CCN3	0.82	2.13	2.81				
IL-1 R6/IL-1 Rrp2	0.76	2.11	2.60				
Frizzled-7	0.56	2.10	2.76				
Vasorin	0.58	2.10	3.78				
Artemin	0.67	2.09	3.64				
IL-20 R alpha	0.56	2.09	3.12				
EDG-1	0.69	2.09	3.71				
Frizzled-1(FZD1)	0.66	2.06	3.03				
TGF-beta 5	0.59	2.06	3.11				
Activin RIB/ALK-4	0.63	2.06	3.51				
CCL28/VIC	0.70	2.06	3.28				
SAA	0.63	2.05	2.95				
Fractalkine	0.96	2.05	3.27				
Activin C	0.70	2.03	2.12				
SMDF/NRG1 Isoform	0.61	2.03	2.91				
TECK/CCL25	0.67	2.02	3.34				
IL-22 R	0.60	2.02	3.03				
E-Selectin	0.59	2.02	3.36				
LRP-1	0.73	2.01	3.45				
HGF	0.56	2.00	2.76				
Endothelin	0.56	2.00	3.58				
TIMP-1	0.21	2.00	9.66				

(Left) 57 growth factors were significantly upregulated and (Right) 35 growth factors were significantly downregulated in P-CM normalized to raw medium. The ratio of P-CM to CM is shown in each part. Abbreviations: MIF, Migration inhibitory factor; IL-18 R alpha, Interleukin 18 receptor alpha; EDA-A2, Ectodysplasin A2; IFN-alpha/beta R1, Interferon-alpha/beta receptor 1; CCR1, CC-Chemokine receptor-1; MDC, Macrophage-derived Chemokine; IGFBP-rp1, Human Insulin-like growth factor binding protein-related protein-1; LECT2, Leukocyte cell-derived chemotaxin-2; Glut5, Glucose transporter 5; GDF8, Growth differentiation factor 8; MIP-1d, Macrophage Inflammatory Protein 1d; VEGF-B, Vascular endothelial growth factor B; TNFRSF19, Tumor necrosis factor receptor superfamily, member 19; FGF-18, Fibroblast growth factor-18; IL-16, Interleukin 16; HGFR, Hepatocyte growth factor receptor; Glut3, Glucose transporter 3; Csk, C-Terminal Src Kinase; TRADD, Tumor necrosis factor receptor type 1-associated death domain; IL-10 R alpha, Interleukin 10 receptor alpha; GITR Ligand, Glucocorticoid-induced Tumor necrosis factor receptor-related protein ligand; GH, Growth Hormone; CTLA-4, Cytotoxic T-lymphocyte-associated protein 4; IL-1 sRI, Interleukin-1 soluble receptor type 1; HVEM, Herpesvirus entry mediator; IL-17C, Interleukin 17C; IL-1 sRII, Interleukin-1 soluble receptor type 2; IL-9, Interleukin-9; CCN3, Cellular communication network factor 3; IL-1 R6, Human Interleukin 1 receptor 6; IL-20 R alpha, Interleukin 20 receptor alpha; EDG-1, Endothelial Differentiation Gene-1; FZD1, Frizzled-1; TGF-beta 5, Transforming growth factor-beta-5; Activin RIB, Activin receptor IB; CCL28, CC-Chemokine ligand 28; SAA, Serum Amyloid A; SMDF, Neuregulin 1 Isoform; TECK, Thymus-expressed Chemokine; IL-22 R, Interleukin 22 receptor; LRP-1, Low-density lipoprotein receptor-related protein 1; HGF, Hepatocyte growth factor; TIMP-1, Tissue inhibitor of metalloproteinases-1; PlGF, Placental growth factor; GCP-2, Granulocyte Chemotactic Protein 2; EDAR, Ectodysplasin receptor; NeuroD1, Neurogenic differentiation 1; IL-1 R8, Human Interleukin 1 receptor 8; LBP, Lipopolysaccharide-binding protein; GDF1, Growth differentiation factor 1; SCF R, Stem cell factor receptor; NCAM-1, Neural cell adhesion molecule 1; MMP-19, Matrix Metalloproteinase 19; Smad 5, Human mothers against DPP homolog 5; PDGF-BB, Platelet-derived growth factor BB; CD14, Cluster of Differentiation 14; sFRP-1, Secreted frizzled-related protein 1; PF4, Platelet Factor 4; MMP-13, Matrix Metalloproteinase 13; NRG1 Isoform GGF2, Neuregulin-1 Isoform glial growth factor 2; TWEAK R, Tumor necrosis factor-related Weak inducer of Apoptosis Receptor; Tie-2, TEK tyrosine kinase; VEGF R3, Vascular endothelial growth factor receptor 3; MCP-3, Monocyte chemoattractant protein-3; MMP-9, Matrix Metalloproteinase 9; PDGF-D, Platelet-derived growth factor D; TCCR, T-Cell Cytokine Receptor; XEDAR, X-linked ectodysplasin-A2 receptor; WISP-1, Wnt Inducible Signaling Pathway Protein 1; MIP-3 alpha, Macrophage Inflammatory Protein 3 Alpha; SCF, Stem cell factor; RANTES, regulated on activation, normal T cell expressed and secreted; ICAM-3, Intercellular Adhesion Molecule 3; LRP-6, Low-density lipoprotein receptor-related protein 6; NT-4, Neurotrophin factor 4.

## Supplementary Materials

The following are available online at https://www.mdpi.com/2073-4409/9/6/1344/s1: Figure S1: Diagram showing the collection of P-CM secreted by hUCB-MSCs; Figure S2: Growth factor array map for the secretion from P-CM-treated DPCs; Figure S3: Western blot analysis of DPCs; Figure S4: Characteristics of mesenchymal stromal cells before and after priming; Figure S5: Clinical study of hair growth with P-CM treatment at 24 weeks; Figure S6: Treatment of DPCs with CM and P-CM for 48 h increases the cell proliferation, as detected by the BrdU Cell proliferation assay; Table S1: Growth factor expression profiling of DPC treated with P-CM; Table S2: Growth factor expression profiling of P-CM; Data S1: Skin Primary Irritation Test in Humans.
