# Supplementary material for: Migration Inhibitory Factor in Conditioned Medium from Human Umbilical Cord Blood-Derived Mesenchymal Stromal Cells Stimulates Hair Growth
Source: Cells. 2020 May 28;9(6):1344. doi: 10.3390/cells9061344 (PMC7349163; doi:10.3390/cells9061344)
Supplement: Supplementary file 1 [file cells-09-01344-s001.zip › Supplemental figure.docx]

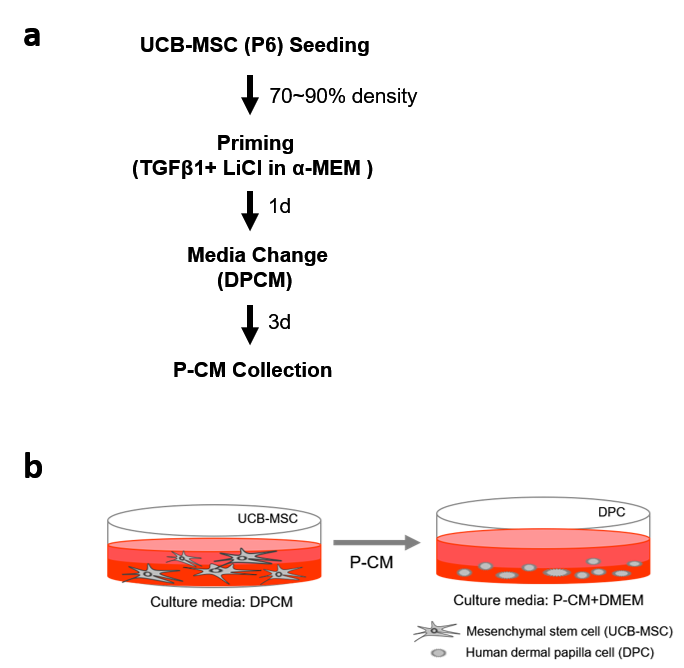


**Figure S1**. Diagram showing the collection of P-CM secreted by hUCB-MSCs. (a) TGFβ1 and LiCl in α-MEM were primed on MSCs for 1day. P-CM was collected after media change to DPCM at 3 days. (b) Collected P-CM was treated on DPCs to determine the cell viability and gene expressions.


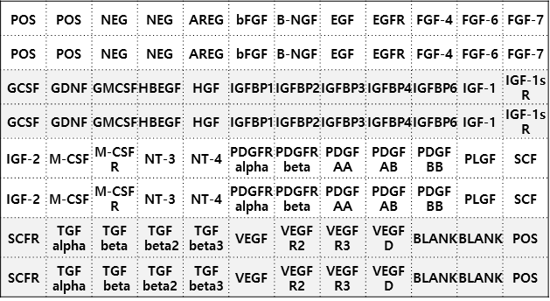


**Figure S2**. Growth factor array map for the secretion from P-CM treated DPCs.

**p-AKT**

**a**

**b**

**Cyclin D1**

**Figure S3**. Western blot analysis of DPCs. There was no significant change of p-AKT and Cyclin D1 expression between CM and P-CM.


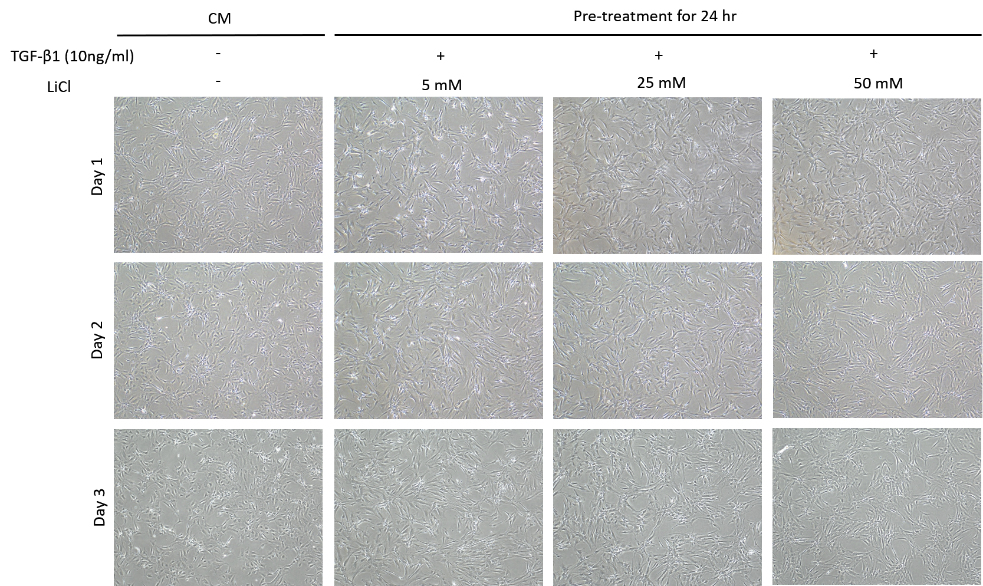


**a**


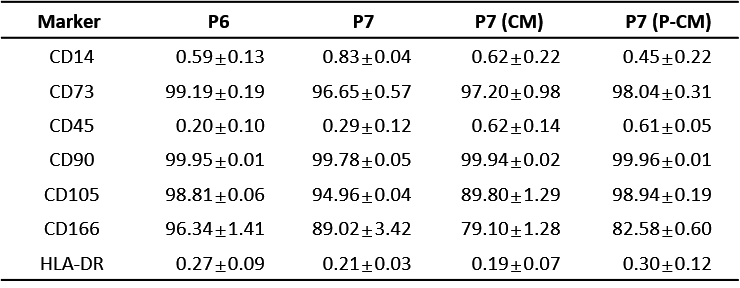


**b**

**c**

**e**

**d**

**Figure S4**. Characteristics of mesenchymal stromal cells (MSCs) before and after priming. (a) MSC morphology was not changed after priming. (b) MSC features were analyzed by representative MSC cell surface marker expression levels (%) using flow cytometry (Positive: CD73, CD90, CD105, CD166 ≥ 80%; Negative: CD14, CD45, HLA-DR ≤ 1.0%). Priming with TGFβ1 and LiCl didn’t change the characteristics of stem cell capacity. (c) The viability of MSCs didn’t show a significant difference after the treatment of TGFβ1 and LiCl. (d) The cell numbers after priming were quantified by direct cell counting. Both groups had no significant difference. (e) MSCs (P7) were seeded at 5000 cells/cm^2^ , and then TGFβ1 and LiCl were treated for 1 d. After 3 days, BrdU solution was added into each well and MSCs were further incubated for 2 h. Optical signal intensity was measured at 370 nm using a VERSAmax microplate reader (Molecular Devices, San Jose, CA, USA). The signal intensity of MSCs priming with TGFβ1 and LiCl had no significant changes compared to no primed MSCs.


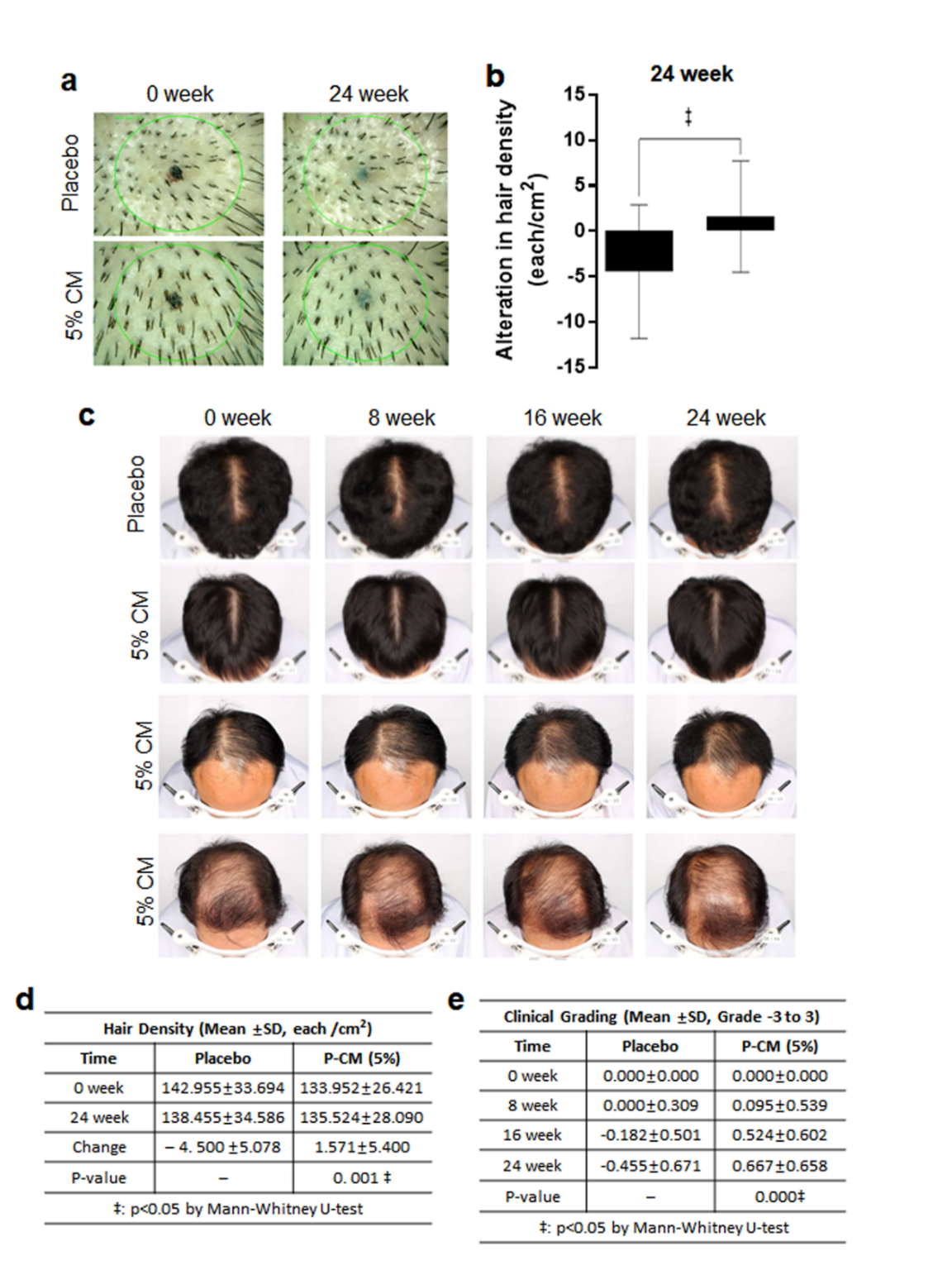


**Figure S5**. Clinical study of hair growth with P-CM treatment at 24 weeks. (a) Comparison between placebo treated group and 5% CM treated group at 24 weeks. (b, c, d) The hair density of 5% CM treated group (right) was increased compared to placebo group (left) at 24 weeks. (e) Clinical grading was determined by professional visual assessment using a phototrichogram. Compared to the patient baseline data (baseline) after 8, 16, and 24 weeks of treatment, the hair growth monitored and visually scored: +2, good; +1, moderate; 0, initial state; -1, poor; -2, bad. Hair density (each/cm^2^) was calculated by counting the total number of hairs in the target area. Total 43 patients (P-CM = 25, placebo = 18) were participated in 24-week clinical trial. Statistically significant differences between the placebo and 5% P-CM were determined by Mann--Whiteney U test. Statistical analysis was performed by Mann-Whitney U-test for unpaired samples. Standard deviations are indicated.

**Figure S6**. Treatment of DPCs with CM and P-CM for 48 h increases cell proliferation as detected by BrdU Cell proliferation assay (Roche, cat# 11647229001, Mannheim, Germany). DPCs were seeded at 4000 cells/well in a 96-well plate and incubated overnight. P-CM was then added into the plate at different concentrations and DPCs were incubated for 48 h. BrdU solution was added into each well and DPCs were further incubated for 2 h. Optical signal intensity was measured at 370 nm using a VERSAmax microplate reader (Molecular Devices, San Jose, CA, USA).
