# Supplementary material for: Migration Inhibitory Factor in Conditioned Medium from Human Umbilical Cord Blood-Derived Mesenchymal Stromal Cells Stimulates Hair Growth
Source: Cells. 2020 May 28;9(6):1344. doi: 10.3390/cells9061344 (PMC7349163; doi:10.3390/cells9061344)
Supplement: Supplementary file 1 [file cells-09-01344-s001.zip › Supplemental Table.docx]

**Supplemental Table 1**. Growth factor expression profiling from DPC treated with P-CM

|  | **Probe name** | **Full Name** | **P-CM** | **DPC + P-CM** |
| --- | --- | --- | --- | --- |
| 1 | AR | Amphiregulin | 1.00 | 0.84 |
| 2 | bFGF | Basic fibroblast growth factor | 1.00 | 0.96 |
| 3 | beta-NGF | Nerve growth factor-beta | 1.00 | 0.99 |
| 4 | EGF | Epidermal growth factor | 1.00 | 1.02 |
| 5 | EGFR | Epidermal growth factor receptor | 1.00 | 1.23 |
| 6 | FGF-4 | Fibroblast growth factor 4 | 1.00 | 1.23 |
| 7 | FGF-6 | Fibroblast growth factor 6 | 1.00 | 1.20 |
| 8 | FGF-7(KGF) | Fibroblast growth factor 7 | 1.00 | 1.18 |
| 9 | GCSF | Granulocyte-colony Stimulating Factor | 1.00 | 0.78 |
| 10 | GDNF | Glial-derived Neurotrophic Factor | 1.00 | 0.70 |
| 11 | GM-CSF | Granulocyte-macrophage colony stimulating factor | 1.00 | 0.76 |
| 12 | HB-EGF | Heparin-binding Epidermal Growth factor | 1.00 | 0.79 |
| 13 | HGF | Hepatocyte growth factor | 1.00 | 0.78 |
| 14 | IGFBP-1 | Insulin-like growth factor binding proteins 1 | 1.00 | 1.03 |
| 15 | IGFBP-2 | Insulin-like growth factor binding proteins 2 | 1.00 | 1.10 |
| 16 | IGFBP-3 | Insulin-like growth factor binding proteins 3 | 1.00 | 1.21 |
| 17 | IGFBP-4 | Insulin-like growth factor binding proteins 4 | 1.00 | 1.10 |
| 18 | IGFBP-6 | Insulin-like growth factor binding proteins 6 | 1.00 | 1.71 |
| 19 | IGF-1 | Insulin growth factor 1 | 1.00 | 0.93 |
| 20 | IGF-1R | Insulin growth factor 1 receptor | 1.00 | 0.94 |
| 21 | IGF-2 | Insulin growth factor 2 | 1.00 | 0.90 |
| 22 | M-CSF | Macrophage-colony Stimulating Factor | 1.00 | 0.92 |
| 23 | M-CSF R | Macrophage-colony Stimulating Factor receptor | 1.00 | 0.92 |
| 24 | NT-3 | Neurotrophin factor 3 | 1.00 | 1.01 |
| 25 | NT-4 | Neurotrophin factor 4 | 1.00 | 1.14 |
| 26 | PDGF R alpha | Platelet-derived Growth Factor receptor alpha | 1.00 | 1.17 |
| 27 | PDGF R beta | Platelet-derived Growth Factor receptor beta | 1.00 | 1.36 |
| 28 | PDGF-AA | Platelet-derived growth factor AA | 1.00 | 0.94 |
| 29 | PDGF-AB | Platelet-derived growth factor AB | 1.00 | 1.16 |
| 30 | PDGF-BB | Platelet-derived growth factor BB | 1.00 | 1.14 |
| 31 | PlGF | Placental growth factor | 1.00 | 1.30 |
| 32 | SCF | Stem cell factor | 1.00 | 1.24 |
| 33 | SCF R (CD117/c-kit) | Stem cell factor receptor | 1.00 | 0.92 |
| 34 | TGF alpha | Transforming growth factor alpha | 1.00 | 1.04 |
| 35 | TGF beta 1 | Transforming growth factor beta 1 | 1.00 | 1.20 |
| 36 | TGF beta 2 | Transforming growth factor beta 2 | 1.00 | 1.23 |
| 37 | TGF beta 3 | Transforming growth factor beta 3 | 1.00 | 1.14 |
| 38 | VEGF-A | Vascular endothelial growth factor A | 1.00 | 1.66 |
| 39 | VEGFR2 | Vascular endothelial growth factor 2 | 1.00 | 1.10 |
| 40 | VEGFR3 | Vascular endothelial growth factor 3 | 1.00 | 1.05 |
| 41 | VEGF-D | Vascular endothelial growth factor D | 1.00 | 1.15 |

**Supplemental Table 2.** Growth factor expression profiling on P-CM

|  | **Probe name** | **Full Name** | **CM/** | **P-CM/** |
| --- | --- | --- | --- | --- |
|  |  |  | **Raw M** | **Raw M** |
| 1 | 6Ckine/CCL21 | C-C motif chemokine 21 | 0.596 | 1.055 |
| 2 | Activin A | Activin A | 1.374 | 1.73 |
| 3 | Activin B | Activin B | 0.718 | 1.507 |
| 4 | Activin C | Activin C | 0.7 | 2.035 |
| 5 | Activin RIA / ALK-2 | Activin receptor IA | 0.633 | 1.472 |
| 6 | Activin RIB / ALK-4 | Activin receptor IB | 0.628 | 2.057 |
| 7 | Activin RII A/B | Activin receptor II | 0.699 | 1.967 |
| 8 | Activin RIIA | Activin receptor IIA | 0.675 | 1.988 |
| 9 | Adiponectin / Acrp30 | Adiponectin | 0.444 | 0.79 |
| 10 | AgRP | Agouti related protein | 0.672 | 1.795 |
| 11 | ALCAM | Adhesion leukocyte cell adhesion molecule | 0.612 | 1.485 |
| 12 | Angiogenin | Angiogenin | 0.461 | 0.413 |
| 13 | Angiopoietin-1 | Angiopoietin-1 | 0.492 | 0.502 |
| 14 | Angiopoietin-2 | Angiopoietin-2 | 0.595 | 0.535 |
| 15 | Angiopoietin-4 | Angiopoietin-4 | 0.538 | 1.543 |
| 16 | Angiopoietin-like 1 | Angiopoietin-like 1 | 0.597 | 1.435 |
| 17 | Angiopoietin-like 2 | Angiopoietin-like 2 | 0.648 | 1.263 |
| 18 | Angiopoietin-like Factor | Angiopoietin-like Factor | 0.732 | 1.794 |
| 19 | Angiostatin | Angiostatin | 0.759 | 2.366 |
| 20 | APJ | Apelin receptor | 0.58 | 1.485 |
| 21 | APRIL/TNFSF13 | Tumor necrosis factor ligand superfamily member 13 | 0.726 | 1.603 |
| 22 | AR (Amphiregulin) | Amphiregulin | 0.702 | 1.98 |
| 23 | Artemin | Artemin | 0.671 | 2.091 |
| 24 | Axl | AXL receptor tyrosine kinase | 0.646 | 1.215 |
| 25 | B7-1 /CD80 | Cluster of Differentiation 80 | 0.311 | 0.851 |
| 26 | BAFF R / TNFRSF13C | TNF receptor superfamily member 13C | 0.576 | 1.567 |
| 27 | BCMA / TNFRSF17 | TNF receptor superfamily member 17 | 0.578 | 1.305 |
| 28 | BD-1 | Beta Defensin-1 | 0.542 | 1.394 |
| 29 | BDNF | Brain-derived neurotrophic factor | 0.569 | 0.591 |
| 30 | beta-Catenin | beta-Catenin | 0.483 | 1.138 |
| 31 | beta-Defensin 2 | Beta-Defensin 2 | 0.597 | 1.374 |
| 32 | beta-NGF | Nerve growth factor-beta | 0.576 | 1.009 |
| 33 | BIK | BCL2 interacting killer | 1.025 | 1.499 |
| 34 | BLC / BCA-1 / CXCL13 | B-lymphocyte chemoattractant | 0.799 | 1.391 |
| 35 | BMP-15 | Bone morphogenetic proteins 15 | 0.514 | 1.664 |
| 36 | BMP-2 | Bone morphogenetic proteins 2 | 0.735 | 1.609 |
| 37 | BMP-3 | Bone morphogenetic proteins 3 | 0.637 | 1.673 |
| 38 | BMP-3b / GDF-10 | Bone morphogenetic proteins 3b | 0.562 | 1.238 |
| 39 | BMP-4 | Bone morphogenetic proteins -4 | 0.736 | 1.339 |
| 40 | BMP-5 | Bone morphogenetic proteins -5 | 0.617 | 1.604 |
| 41 | BMP-6 | Bone morphogenetic proteins -6 | 0.42 | 0.972 |
| 42 | BMP-7 | Bone morphogenetic proteins -7 | 0.605 | 1.912 |
| 43 | BMP-8 | Bone morphogenetic proteins -8 | 0.605 | 1.913 |
| 44 | BMPR-IA / ALK-3 | Bone morphogenetic proteins receptor IA | 0.57 | 0.809 |
| 45 | BMPR-IB / ALK-6 | Bone morphogenetic proteins receptor IB | 0.663 | 0.834 |
| 46 | BMPR-II | Bone morphogenetic proteins receptor II | 0.618 | 0.863 |
| 47 | BTC | Betacellulin | 0.622 | 1.645 |
| 48 | Cardiotrophin-1 / CT-1 | Cardiotrophin-1 | 0.623 | 1.253 |
| 49 | CCL14 / HCC-1 / HCC-3 | CC-Chemokine ligand 14 | 0.7 | 1.43 |
| 50 | CCL28 / VIC | CC-Chemokine ligand 28 | 0.696 | 2.055 |
| 51 | CCR1 | CC-Chemokine receptor-1 | 2.636 | 5.968 |
| 52 | CCR2 | CC-Chemokine receptor-2 | 0.88 | 1.75 |
| 53 | CCR3 | CC-Chemokine receptor-3 | 0.64 | 1.68 |
| 54 | CCR4 | CC-Chemokine receptor-4 | 0.555 | 1.203 |
| 55 | CCR5 | CC-Chemokine receptor-5 | 0.67 | 1.503 |
| 56 | CCR6 | CC-Chemokine receptor-6 | 0.638 | 1.375 |
| 57 | CCR7 | CC-Chemokine receptor-7 | 0.536 | 1.015 |
| 58 | CCR8 | CC-Chemokine receptor-8 | 0.375 | 0.842 |
| 59 | CCR9 | CC-Chemokine receptor-9 | 0.48 | 1.018 |
| 60 | CD 163 | Cluster of Differentiation 163 | 0.601 | 1.503 |
| 61 | CD14 | Cluster of Differentiation 14 | 0.57 | 0.449 |
| 62 | CD27 / TNFRSF7 | Cluster of Differentiation 27 | 0.61 | 0.907 |
| 63 | CD30 / TNFRSF8 | Cluster of Differentiation 30 | 0.66 | 1.939 |
| 64 | CD30 Ligand / TNFSF8 | Cluster of Differentiation 30 ligand | 0.808 | 1.684 |
| 65 | CD40 / TNFRSF5 | Cluster of Differentiation40 | 0.65 | 1.093 |
| 66 | CD40 Ligand / TNFSF5 /CD154 | Cluster of Differentiation 40 ligand | 0.702 | 1.638 |
| 67 | Cerberus 1 | Cerberus 1 | 1.437 | 2.788 |
| 68 | Chem R23 | Chemokine like receptor 1 | 0.535 | 1.437 |
| 69 | Chordin-Like 1 | Chordin-Like 1 | 0.399 | 1.068 |
| 70 | Chordin-Like 2 | Chordin-Like 2 | 0.407 | 1.385 |
| 71 | CLC | Cardiotrophin-like cytokine | 0.491 | 1.45 |
| 72 | CNTF R alpha | Ciliary neurotrophic factor receptor alpha | 0.665 | 1.962 |
| 73 | CNTF | Ciliary neuronotrophic factor | 0.639 | 1.768 |
| 74 | Coagulation Factor III / Tissue Factor | Coagulation Factor III | 0.436 | 0.808 |
| 75 | CRIM 1 | Cysteine-rich Motor Neuron 1 | 0.603 | 1.42 |
| 76 | Cripto-1 | Cripto-1 growth factor | 0.643 | 1.162 |
| 77 | CRTH-2 | Chemoattrraactant receptor-homologous molecule expressed on Th2 cells | 0.586 | 1.485 |
| 78 | Cryptic | Cryptic | 0.722 | 1.788 |
| 79 | Csk | C-Terminal Src Kinase | 1.212 | 2.21 |
| 80 | CTACK / CCL27 | Cutaneous T-Cell Attracting Chemokine | 0.683 | 1.856 |
| 81 | CTGF / CCN2 | Connective Tissue Growth Factor | 0.704 | 1.658 |
| 82 | CTLA-4 /CD152 | Cytotoxic T-lymphocyte associated protein 4 | 0.693 | 2.149 |
| 83 | CV-2 / Crossveinless-2 | Crossveinless-2 | 0.64 | 2.504 |
| 84 | CXCL14 / BRAK | CXC Chemokine ligand 14 | 0.509 | 0.981 |
| 85 | CXCL16 | CXC Chemokine ligand 16 | 0.325 | 0.775 |
| 86 | CXCR1 / IL-8 RA | CXC-Chemokine receptor 1 | 0.534 | 1.357 |
| 87 | CXCR2 / IL-8 RB | CXC-Chemokine receptor 2 | 0.576 | 1.091 |
| 88 | CXCR3 | CXC-Chemokine receptor 3 | 0.786 | 1.739 |
| 89 | CXCR4 (fusin) | CXC-Chemokine receptor 4 | 0.634 | 1.371 |
| 90 | CXCR5 /BLR-1 | CXC-Chemokine receptor 5 | 0.679 | 1.109 |
| 91 | CXCR6 | CXC-Chemokine receptor 6 | 0.55 | 0.838 |
| 92 | D6 | Atypical chemokine receptor 2 | 0.517 | 1.243 |
| 93 | DAN | Differential screening-selected gene aberrant in neuroblastoma | 0.754 | 1.162 |
| 94 | DANCE | Developmental Arteries and Neural CrestEGF like protein | 0.488 | 1.084 |
| 95 | DcR3 / TNFRSF6B | Decoy Receptor 3 | 0.613 | 1.202 |
| 96 | Decorin | Decorin | 0.72 | 1.273 |
| 97 | Dkk-1 | Dickkopf-related protein 1 | 0.749 | 1.33 |
| 98 | Dkk-3 | Dickkopf-related protein 3 | 0.77 | 1.634 |
| 99 | Dkk-4 | Dickkopf-4 | 0.59 | 1.152 |
| 100 | DR3 / TNFRSF25 | TNF receptor superfamily member 25 | 0.373 | 0.77 |
| 101 | DR6 / TNFRSF21 | Death receptor-6 /TNF receptor superfamily member 21 | 0.565 | 1.762 |
| 102 | Dtk | Tyrosine-protein kinase receptor TYRO3 | 0.466 | 0.82 |
| 103 | EDA-A2 | Ectodysplasin A | 9.721 | 8.095 |
| 104 | EDAR | Ectodysplasin receptor | 0.04 | 0.5 |
| 105 | EDG-1 | Endothelial Differentiation Gene-1 | 0.689 | 2.087 |
| 106 | EGF | Epidermal growth factor | 0.396 | 0.595 |
| 107 | EGF R / ErbB1 | Epidermal growth factor receptor | 0.415 | 0.802 |
| 108 | EG-VEGF / PK1 | Endocrine Gland-derived Vascular Endothelial cell Growth Factor | 0.622 | 1.646 |
| 109 | EMAP-II | Endothelial Monocyte-Activation Polypeptide II | 0.65 | 1.808 |
| 110 | ENA-78 | Epithelial neutrophil-activating protein 78 | 0.831 | 1.501 |
| 111 | Endocan | Endocan | 0.559 | 1.478 |
| 112 | Endoglin / CD105 | Endoglin | 0.658 | 1.841 |
| 113 | Endostatin | Collagen type XVIII alpha 1 chain | 0.637 | 1.774 |
| 114 | Endothelin | Endothelin | 0.558 | 1.998 |
| 115 | EN-RAGE | S100 calcium binding protein A12 | 0.327 | 0.72 |
| 116 | Eotaxin / CCL11 | Eotaxin-1 | 0.226 | 1.059 |
| 117 | Eotaxin-2 / MPIF-2 | Eotaxin-2 | 0.833 | 1.442 |
| 118 | Eotaxin-3 / CCL26 | Eotaxin-3 | 0.674 | 1.461 |
| 119 | Epiregulin | Epiregulin | 0.344 | 0.713 |
| 120 | ErbB2 | Erb-b2 receptor tyrosine kinase 2 | 0.727 | 1.288 |
| 121 | ErbB3 | Erb-b2 receptor tyrosine kinase 3 | 1 | 0.601 |
| 122 | ErbB4 | Erb-b2 receptor tyrosine kinase 4 | 0.621 | 0.98 |
| 123 | Erythropoietin | Erythropoietin | 0.623 | 1.977 |
| 124 | E-Selectin | E-selectin | 0.586 | 2.017 |
| 125 | FADD | Fas-Associating protein with Death Domain | 0.603 | 1.118 |
| 126 | FAM3B | Family with sequence similarity 3 member B | 0.715 | 1.387 |
| 127 | Fas / TNFRSF6 | Tumor necrosis factor receptor superfamily member 6 | 1.331 | 1.848 |
| 128 | Fas Ligand | Tumor necrosis factor ligand 1A | 0.662 | 1.552 |
| 129 | FGF Basic | Basic fibroblast growth factor | 0.381 | 1.112 |
| 130 | FGF R3 | Fibroblast growth factor receptor 3 alpha | 0.543 | 1.55 |
| 131 | FGF R4 | Fibroblast growth factor receptor 4 | 0.583 | 1.852 |
| 132 | FGF R5 | Fibroblast growth factor receptor 5 | 0.574 | 1.164 |
| 133 | FGF-10 / KGF-2 | Fibroblast growth factor-10 | 0.707 | 1.711 |
| 134 | FGF-11 | Fibroblast growth factor-11 | 0.71 | 1.931 |
| 135 | FGF-12 | Fibroblast growth factor-12 | 0.6 | 1.49 |
| 136 | FGF-13 1B | Fibroblast growth factor-13 isoform 1B | 0.427 | 1.838 |
| 137 | FGF-16 | Fibroblast growth factor-16 | 0.539 | 1.69 |
| 138 | FGF-17 | Fibroblast growth factor-17 | 0.245 | 1.474 |
| 139 | FGF-18 | Fibroblast growth factor-18 | 0.511 | 2.689 |
| 140 | FGF-19 | Fibroblast growth factor-19 | 0.503 | 1.631 |
| 141 | FGF-20 | Fibroblast growth factor-20 | 0.419 | 1.781 |
| 142 | FGF-21 | Fibroblast growth factor-21 | 0.263 | 0.735 |
| 143 | FGF-23 | Fibroblast growth factor-23 | 0.484 | 1.121 |
| 144 | FGF-4 | Fibroblast growth factor-4 | 0.853 | 0.975 |
| 145 | FGF-5 | Fibroblast growth factor-5 | 0.679 | 1.597 |
| 146 | FGF-6 | Fibroblast growth factor-6 | 0.375 | 0.729 |
| 147 | FGF-7 / KGF | Fibroblast growth factor-7 | 0.441 | 0.772 |
| 148 | FGF-8 | Fibroblast growth factor-8 | 0.312 | 1.193 |
| 149 | FGF-9 | Fibroblast growth factor-9 | 0.388 | 1.361 |
| 150 | FGF-BP | Fibroblast growth factor-binding protein | 0.571 | 1.937 |
| 151 | FLRG | Follistatin-Related gene protein | 0.702 | 1.501 |
| 152 | Flt-3 Ligand | Fms-like tyrosine kinase receptor 3 Ligand | 0.611 | 1.87 |
| 153 | Follistatin | Follistatin | 0.633 | 1.572 |
| 154 | Follistatin-like 1 | Follistatin-like 1 | 0.548 | 1.35 |
| 155 | Fractalkine | Fractalkine | 0.964 | 2.046 |
| 156 | Frizzled-1 | Frizzled-1 | 0.663 | 2.063 |
| 157 | Frizzled-3(FZD3) | Frizzled-3 | 0.664 | 2.883 |
| 158 | Frizzled-4 | Frizzled-4 | 0.304 | 1.286 |
| 159 | Frizzled-5 | Frizzled-5 | 0.241 | 0.804 |
| 160 | Frizzled-6 | Frizzled-6 | 0.339 | 1.34 |
| 161 | Frizzled-7 | Frizzled-7 | 0.557 | 2.103 |
| 162 | Galectin-3 | Galectin-3 | 0.189 | 0.845 |
| 163 | GASP-1 / WFIKKNRP | G-protein coupled receptor-associated sorting protein 1 | 0.592 | 1.906 |
| 164 | GASP-2 / WFIKKN | G-protein coupled receptor-associated sorting protein 2 | 0.548 | 1.812 |
| 165 | GCP-2 / CXCL6 | Granulocyte Chemotactic Protein 2 | 0.075 | 0.5 |
| 166 | GCSF | Granulocyte-colony Stimulating Factor | 0.649 | 1.565 |
| 167 | G-CSF R / CD 114 | Granulocyte-colony Stimulating Factor receptor | 0.6 | 1.142 |
| 168 | GDF1 | Growth differentiation factor 1 | 0.4 | 0.471 |
| 169 | GDF11 | Growth differentiation factor 11 | 0.597 | 1.698 |
| 170 | GDF-15 | Growth differentiation factor 15 | 0.649 | 1.369 |
| 171 | GDF3 | Growth differentiation factor 3 | 0.734 | 1.321 |
| 172 | GDF5 | Growth differentiation factor 5 | 0.734 | 1.412 |
| 173 | GDF8 | Growth differentiation factor 8 | 0.783 | 3.107 |
| 174 | GDF9 | Growth differentiation factor 9 | 0.673 | 1.647 |
| 175 | GDNF | Glial-derived Neurotrophic Factor | 0.321 | 1.346 |
| 176 | GFR alpha-1 | GDNF Family Receptor alpha 1 | 0.263 | 1.208 |
| 177 | GFR alpha-2 | Glial cell line derived neurotrophic factor alpha 2 | 0.566 | 1.893 |
| 178 | GFR alpha-3 | GDNF family receptor alpha 3 | 0.634 | 1.599 |
| 179 | GFR alpha-4 | GDNF family receptor alpha 4 | 0.571 | 1.348 |
| 180 | GITR / TNFRF18 | Glucocorticoid-induced TNFR-related protein | 0.705 | 1.733 |
| 181 | GITR Ligand / TNFSF18 | Glucocorticoid-induced TNFR-related protein ligand | 0.682 | 2.194 |
| 182 | Glucagon | Glucagon | 0.867 | 1.811 |
| 183 | Glut1 | glucose transporter 1 | 0.564 | 1.666 |
| 184 | Glut2 | glucose transporter 2 | 0.465 | 1.232 |
| 185 | Glut3 | Glucose transporter 3 | 0.616 | 2.276 |
| 186 | Glut5 | Glucose transporter 5 | 0.594 | 3.185 |
| 187 | Glypican 3 | Glypican 3 | 0.641 | 2.75 |
| 188 | Glypican 5 | Glypican 5 | 2.152 | 1.763 |
| 189 | GM-CSF | Granulocyte-macrophage colony stimulating factor | 0.654 | 1.803 |
| 190 | GM-CSF R alpha | Granulocyte macrophage colony stimulating factor receptor alpha | 0.457 | 1.283 |
| 191 | Granzyme A | Granzyme A | 0.617 | 1.742 |
| 192 | GREMLIN | gremlin 1 | 0.729 | 1.262 |
| 193 | GRO | Growth Related Oncogene | 0.751 | 1.682 |
| 194 | GRO-a | Growth Related Oncogene-Alpha | 0.696 | 1.143 |
| 195 | Growth Hormone (GH) | Growth Hormone | 0.754 | 2.175 |
| 196 | Growth Hormone R (GHR) | Growth Hormone Receptor | 0.476 | 1.046 |
| 197 | HB-EGF | Heparin-binding Epidermal Growth factor | 0.596 | 1.311 |
| 198 | HCC-4 / CCL16 | Hemofiltrate CC Chemokine 4 | 0.914 | 1.252 |
| 199 | HCR / CRAM-A/B | Human Chemokine Receptor CRAM-A isoform | 0.573 | 1.365 |
| 200 | Hepassocin | Hepassocin | 0.628 | 2.17 |
| 201 | Heregulin / NDF / GGF / Neuregulin | Neuregulin | 0.725 | 1.549 |
| 202 | HGF | Hepatocyte growth factor | 0.555 | 1.999 |
| 203 | HGFR | Hepatocyte growth factor receptor | 1.046 | 2.289 |
| 204 | HRG-alpha | Heregulin-α | 0.42 | 1.852 |
| 205 | HRG-beta 1 | Heregulin-β1 | 0.086 | 0.751 |
| 206 | HVEM / TNFRSF14 | Herpesvirus entry mediator | 0.735 | 2.146 |
| 207 | I-309 | C-C motif chemokine 1 | 0.658 | 1.011 |
| 208 | ICAM-1 | Intercellular Adhesion Molecule 1 | 0.592 | 0.793 |
| 209 | ICAM-2 | Intercellular Adhesion Molecule 3 | 0.2 | 0.884 |
| 210 | ICAM-3 (CD50) | Intercellular Adhesion Molecule 3 | 0.118 | 0.139 |
| 211 | ICAM-5 | Human Intercellular Adhesion Molecule 5 | 0.587 | 1.619 |
| 212 | IFN-alpha / beta R1 | Interferon alpha/beta receptor 1 | 14.497 | 7.656 |
| 213 | IFN-alpha / beta R2 | Interferon alpha/beta receptor 2 | 0.915 | 0.937 |
| 214 | IFN-beta | interferon beta | 0.592 | 0.984 |
| 215 | IFN-gamma | interferon gamma | 0.685 | 1.517 |
| 216 | IFN-gamma R1 | Interferon gamma receptor 1 | 0.24 | 1.026 |
| 217 | IGFBP-1 | Insulin-like growth factor binding proteins 1 | 0.584 | 1.74 |
| 218 | IGFBP-2 | Insulin-like growth factor binding proteins 2 | 0.471 | 1.18 |
| 219 | IGFBP-3 | Insulin-like growth factor binding proteins 3 | 0.327 | 1.075 |
| 220 | IGFBP-4 | Insulin-like growth factor binding proteins 4 | 0.15 | 0.931 |
| 221 | IGFBP-6 | Insulin-like growth factor binding proteins 6 | 0.281 | 1.637 |
| 222 | IGFBP-rp1 / IGFBP-7 | Human Insulin-like growth factor binding protein-related protein-1 | 1.898 | 3.58 |
| 223 | IGF-I | Insulin-like growth factor-1 | 0.717 | 1.073 |
| 224 | IGF-I SR | Insulin-like growth factor-1 receptor | 0.591 | 1.399 |
| 225 | IGF-II | Insulin-like growth factor-2 | 0.331 | 1.109 |
| 226 | IGF-II R | Human Insulin-like growth factor II receptor | 0.087 | 0.819 |
| 227 | IL-1 alpha | Interleukin I Alpha | 0.711 | 1.798 |
| 228 | IL-1 beta | Interleukin I beta | 1.262 | 1.062 |
| 229 | IL-1 F10 / IL-1HY2 | Human Interleukin 1 family member 10 | 0.375 | 1.456 |
| 230 | IL-1 F5 / FIL1delta | Human Interleukin 1 family member 5 | 0.396 | 0.891 |
| 231 | IL-1 F6 / FIL1 epsilon | Human Interleukin 1 family member 6 | 0.416 | 1.434 |
| 232 | IL-1 F7 / FIL1 zeta | Human Interleukin 1 family member 7 | 0.389 | 1.204 |
| 233 | IL-1 F8 / FIL1 eta | Human Interleukin 1 family member 8 | 0.219 | 1.162 |
| 234 | IL-1 F9 / IL-1 H1 | Human Interleukin 1 family member 9 | 0.577 | 1.41 |
| 235 | IL-1 R3 / IL-1 R AcP | Human Interleukin 1 receptor 3 | 0.258 | 1.592 |
| 236 | IL-1 R4 / ST2 | Human Interleukin 1 receptor 4 | 0.643 | 1.417 |
| 237 | IL-1 R6 / IL-1 Rrp2 | Human Interleukin 1 receptor 6 | 0.764 | 2.108 |
| 238 | IL-1 R8 | Human Interleukin 1 receptor 8 | 0.197 | 0.49 |
| 239 | IL-1 R9 | Human Interleukin 1 receptor superfamily member 9 | 0.272 | 0.966 |
| 240 | IL-1 ra | IL1 receptor antagonist | 0.72 | 1.915 |
| 241 | IL-1 sRI | Interleukin-1 soluble receptor type 1 | 0.641 | 2.149 |
| 242 | IL-1 sRII | Interleukin-1 soluble receptor type 2 | 0.675 | 2.137 |
| 243 | IL-10 | Interleukin 10 | 0.735 | 1.676 |
| 244 | IL-10 R alpha | Interleukin 10 receptor alpha | 0.551 | 2.207 |
| 245 | IL-10 R beta | Interleukin 10 receptor beta | 0.618 | 1.579 |
| 246 | IL-11 | Interleukin 11 | 0.467 | 1.256 |
| 247 | IL-12 p40 | Interleukin 12 P40 | 0.459 | 1.027 |
| 248 | IL-12 p70 | Interleukin 12 p70 | 0.629 | 1.976 |
| 249 | IL-12 R beta 1 | Interleukin 12 receptor beta 1 | 0.663 | 1.232 |
| 250 | IL-12 R beta 2 | Interleukin 12 receptor beta 2 | 0.29 | 0.997 |
| 251 | IL-13 | Interleukin 13 | 0.668 | 1.514 |
| 252 | IL-13 R alpha 1 | Interleukin 13 receptor alpha 1 | 0.808 | 1.132 |
| 253 | IL-13 R alpha 2 | Interleukin 13 receptor alpha 2 | 0.667 | 0.915 |
| 254 | IL-15 | Interleukin 15 | 0.791 | 1.449 |
| 255 | IL-15 R alpha | Interleukin 15 receptor alpha | 0.245 | 0.737 |
| 256 | IL-16 | Interleukin 16 | 1.826 | 2.686 |
| 257 | IL-17 | Interleukin 17 | 0.64 | 1.939 |
| 258 | IL-17B | Interleukin 17B | 0.769 | 0.801 |
| 259 | IL-17B R | Interleukin 17B receptor | 0.522 | 0.982 |
| 260 | IL-17C | Interleukin 17C | 0.096 | 2.145 |
| 261 | IL-17D | Interleukin 17D | 0.316 | 1.179 |
| 262 | IL-17E | Interleukin 17E | 0.383 | 1.104 |
| 263 | IL-17F | Interleukin 17F | 0.177 | 0.625 |
| 264 | IL-17R | Interleukin 17 receptor | 0.617 | 1.247 |
| 265 | IL-17RC | Interleukin 17 receptor C | 0.453 | 0.846 |
| 266 | IL-17RD | Interleukin 17 receptor D | 0.432 | 1.376 |
| 267 | IL-18 BPa | Interleukin 18 Binding protein a | 0.951 | 1.054 |
| 268 | IL-18 R alpha /IL-1 R5 | Interleukin 18 receptor alpha | 3.874 | 8.902 |
| 269 | IL-18 R beta /AcPL | Interleukin 18 receptor beta | 0.93 | 0.925 |
| 270 | IL-19 | Interleukin 19 | 0.389 | 1.113 |
| 271 | IL-2 | Interleukin 2 | 0.752 | 1.548 |
| 272 | IL-2 R alpha | Interleukin 2 Receptor alpha | 0.702 | 0.613 |
| 273 | IL-2 R beta /CD122 | Interleukin 2 Receptor beta | 0.68 | 1.742 |
| 274 | IL-2 R gamma | Interleukin 2 Receptor gamma | 0.529 | 1.16 |
| 275 | IL-20 | Interleukin 20 | 0.247 | 0.996 |
| 276 | IL-20 R alpha | Interleukin 20 receptor alpha | 0.563 | 2.09 |
| 277 | IL-20 R beta | Interleukin 20 receptor beta | 0.308 | 0.929 |
| 278 | IL-21 | Interleukin-21 | 0.571 | 1.442 |
| 279 | IL-21 R | Interleukin 21 receptor | 0.693 | 1.112 |
| 280 | IL-22 | Interleukin-22 | 0.623 | 1.086 |
| 281 | IL-22 BP | Interleukin-22 binding protein | 0.844 | 0.989 |
| 282 | IL-22 R | Interleukin 22 receptor | 0.603 | 2.023 |
| 283 | IL-23 | Interleukin 23 | 0.22 | 1.202 |
| 284 | IL-23 R | Interleukin 23 receptor | 0.534 | 1.141 |
| 285 | IL-24 | Interleukin-24 | 0.504 | 1.014 |
| 286 | IL-26 | Interleukin-26 | 0.624 | 1.1 |
| 287 | IL-27 | Interleukin-27 | 0.454 | 1.109 |
| 288 | IL-28A | Interleukin-28A | 0.328 | 0.814 |
| 289 | IL-29 | Interleukin-29 | 0.271 | 1.089 |
| 290 | IL-3 | Interleukin 3 | 0.559 | 1.894 |
| 291 | IL-3 R alpha | Human Interleukin 3 receptor alpha | 0.522 | 1.459 |
| 292 | IL-31 | Interleukin-31 | 0.439 | 1.862 |
| 293 | IL-31 RA | Interleukin-31 Receptor A | 0.422 | 1.534 |
| 294 | IL-4 | Interleukin 4 | 0.101 | 0.705 |
| 295 | IL-4 R | Interleukin 4 receptor | 0.482 | 1.064 |
| 296 | IL-5 | Interleukin 5 | 0.618 | 1.678 |
| 297 | IL-5 R alpha | Interleukin 5 receptor alpha | 0.613 | 0.912 |
| 298 | IL-6 | Interleukin 6 | 0.817 | 1.758 |
| 299 | IL-6 R | Interleukin 6 receptor | 0.931 | 0.996 |
| 300 | IL-7 | Interleukin 7 | 0.752 | 1.77 |
| 301 | IL-7 R alpha | Interleukin 7 receptor alpha | 0.38 | 0.76 |
| 302 | IL-8 | Interleukin 8 | 0.7 | 1.68 |
| 303 | IL-9 | Interleukin-9 | 0.758 | 2.132 |
| 304 | Inhibin A | Inhibin A | 0.671 | 1.199 |
| 305 | Inhibin B | Inhibin B | 0.425 | 1.029 |
| 306 | Insulin | Insulin | 0.577 | 1.237 |
| 307 | Insulin R | Insulin Receptor | 0.72 | 1.75 |
| 308 | Insulysin / IDE | Insulysin | 0.285 | 0.824 |
| 309 | IP-10 | Interferon-inducible protein-10 | 0.801 | 0.678 |
| 310 | I-TAC / CXCL11 | Interferon-inducible T cell Alpha Chemoattractant | 0.641 | 1.229 |
| 311 | Kininostatin / kininogen | Kininostatin | 0.271 | 0.568 |
| 312 | Kremen-1 | Kremen-1 | 0.507 | 1.63 |
| 313 | Kremen-2 | Kremen-2 | 0.486 | 0.9 |
| 314 | Latent TGF-beta bp1 | Latent TGF-beta bp1 | 0.655 | 1.49 |
| 315 | LBP | Lipopolysaccharide-binding protein | 0.047 | 0.481 |
| 316 | Lck | Lymphocyte-specific protein tyrosine kinase | 0.194 | 1.042 |
| 317 | LECT2 | Leukocyte cell-derived chemotaxin-2 | 0.6 | 3.461 |
| 318 | Lefty - A | Lefty long isoform | 0.452 | 1.451 |
| 319 | Leptin (OB) | Leptin | 0.461 | 0.676 |
| 320 | Leptin R | Leptin Receptor | 0.533 | 1.115 |
| 321 | LFA-1 alpha | lymphocyte function-associated antigen 1 alpha | 0.56 | 0.905 |
| 322 | LIF R alpha | Leukemia Inhibitory Factor Receptor alpha | 0.671 | 1.865 |
| 323 | LIF | Leukemia Inhibitory Factor | 0.865 | 1.272 |
| 324 | LIGHT / TNFSF14 | Tumor necrosis factor superfamily member 14 | 0.805 | 1.088 |
| 325 | Lipocalin-1 | Lipocalin-1 | 0.476 | 0.755 |
| 326 | Lipocalin-2 | Lipocalin-2 | 0.37 | 1.028 |
| 327 | LRP-1 | Low density lipoprotein receptor-related protein 1 | 0.727 | 2.006 |
| 328 | LRP-6 | Low density lipoprotein receptor related protein 6 | 0.016 | 0.134 |
| 329 | L-Selectin (CD62L) | L-selectin | 0.624 | 1.416 |
| 330 | Lymphotactin / XCL1 | Lymphotactin | 0.432 | 0.836 |
| 331 | Lymphotoxin beta / TNFSF3 | Lymphotoxin alpha / beta | 0.29 | 0.655 |
| 332 | Lymphotoxin beta R / TNFRSF3 | Lymphotoxin beta receptor | 0.544 | 1.344 |
| 333 | MAC-1 | Macrophage galactose-specific lectin-1 | 0.58 | 1.733 |
| 334 | MCP-1 | Monocyte Chemoattractant Protein 1 | 0.65 | 1.683 |
| 335 | MCP-2 | Monocyte chemoattractant protein-2 | 0.509 | 0.976 |
| 336 | MCP-3 | Monocyte chemoattractant protein-3 | 0.449 | 0.402 |
| 337 | MCP-4 / CCL13 | Monocyte Chemoattractant Protein 4 | 0.864 | 0.689 |
| 338 | M-CSF | Macrophage-colony Stimulating Factor | 0.653 | 1.161 |
| 339 | M-CSF R | Macrophage-colony Stimulating Factor receptor | 0.489 | 0.891 |
| 340 | MDC | Macrophage-derived Chemokine | 1.555 | 4.65 |
| 341 | MFG-E8 | Milk Fat Globule-EGF Factor 8 Protein | 0.63 | 0.936 |
| 342 | MFRP | Membrane-type Frizzled-related protein | 0.532 | 1.537 |
| 343 | MICA | MHC class I polypeptide–related sequence A | 0.484 | 0.674 |
| 344 | MIF | migration inhibition factor | 5.499 | 22.619 |
| 345 | MIG | monokine induced by gamma interferon | 0.682 | 1.687 |
| 346 | MIP 2 | Macrophage Inflammatory Protein 2 | 0.525 | 1.582 |
| 347 | MIP-1a | Macrophage Inflammatory Protein 1 Alpha | 0.483 | 0.904 |
| 348 | MIP-1b | Macrophage Inflammatory Protein 1 Beta | 0.649 | 1.4 |
| 349 | MIP-1d | Macrophage Inflammatory Protein 1d | 0.804 | 2.954 |
| 350 | MIP-3 alpha | Macrophage Inflammatory Protein 3 Alpha | 0.278 | 0.279 |
| 351 | MIP-3 beta | Macrophage Inflammatory Protein 3 Beta | 0.636 | 0.885 |
| 352 | MMP-1 | Matrix Metalloproteinase 1 | 0.729 | 1.517 |
| 353 | MMP-10 | Matrix Metalloproteinase 10 | 0.861 | 1.108 |
| 354 | MMP-11 /Stromelysin-3 | Matrix Metalloproteinase 11 | 0.479 | 1.549 |
| 355 | MMP-12 | Matrix Metalloproteinase 12 | 0.523 | 0.897 |
| 356 | MMP-13 | Matrix Metalloproteinase 13 | 0.017 | 0.429 |
| 357 | MMP-14 | Matrix Metalloproteinase 14 | 0.548 | 1.348 |
| 358 | MMP-15 | Matrix Metalloproteinase 15 | 0.716 | 1.47 |
| 359 | MMP-16 / MT3-MMP | Matrix Metalloproteinase 16 | 0.572 | 1.359 |
| 360 | MMP-19 | Matrix Metalloproteinase 19 | 1.055 | 0.461 |
| 361 | MMP-2 | Matrix Metalloproteinase 2 | 0.659 | 1.285 |
| 362 | MMP-20 | Matrix Metalloproteinase 20 | 0.797 | 1.106 |
| 363 | MMP-24 / MT5-MMP | Matrix Metalloproteinase 24 | 0.819 | 0.691 |
| 364 | MMP-25 / MT6-MMP | Matrix Metalloproteinase 25 | 0.631 | 1.025 |
| 365 | MMP-3 | Matrix Metalloproteinase 3 | 0.569 | 1.756 |
| 366 | MMP-7 | Matrix Metalloproteinase 7 | 0.327 | 0.897 |
| 367 | MMP-8 | Matrix Metalloproteinase 8 | 0.592 | 1.342 |
| 368 | MMP-9 | Matrix Metalloproteinase 9 | 0.251 | 0.401 |
| 369 | MSP alpha Chain | Macrophage stimulating Protein 1 | 0.554 | 0.893 |
| 370 | Musk | Muscle-Specific Kinase | 1 | 1.179 |
| 371 | NAP-2 | Human neural cell adhesion molecule 1 | 0.053 | 0.773 |
| 372 | NCAM-1 / CD56 | Neural cell adhesion molecule 1 | 0.105 | 0.469 |
| 373 | Neuritin | Neuritin | 0.652 | 1.202 |
| 374 | NeuroD1 | Neurogenic differentiation 1 | 0.287 | 0.494 |
| 375 | Neuropilin-2 | Human Neuropilin-2 | 0.426 | 1.11 |
| 376 | Neurturin | Neurturin | 0.33 | 0.983 |
| 377 | NGF R | nerve growth factor receptor | 0.427 | 1.119 |
| 378 | Nidgen-1 | Nidgen-1 | 0.338 | 0.927 |
| 379 | NOV / CCN3 | Cellular communication network factor 3 | 0.821 | 2.131 |
| 380 | NrCAM | Neuronal Cell Adhesion Molecule | 0.536 | 1.267 |
| 381 | NRG1 Isoform GGF2 | Neuregulin‑1 Isoform glial growth factor 2 | 0.344 | 0.424 |
| 382 | NRG2 | Neuregulin 2 | 0.722 | 0.782 |
| 383 | NRG3 | Neuregulin 3 | 0.69 | 1.435 |
| 384 | NT-3 | Neurotrophin factor 3 | 0.563 | 1.058 |
| 385 | NT-4 | Neurotrophin factor 4 | 0.095 | 0.112 |
| 386 | Orexin A | Orexin A | 0.125 | 0.661 |
| 387 | Orexin B | Orexin B | 0.534 | 1.748 |
| 388 | OSM | Oncostatin M | 0.621 | 1.16 |
| 389 | Osteoactivin / GPNMB | Osteoactivin | 0.521 | 1.029 |
| 390 | Osteocrin | Osteocrin | 0.409 | 1.289 |
| 391 | Osteoprotegerin / TNFRSF11B | Osteoprotegerin | 0.038 | 0.654 |
| 392 | OX40 Ligand / TNFSF4 | Tumor necrosis factor (ligand) superfamily, member 4 | 0.437 | 1.072 |
| 393 | PARC / CCL18 | C-C motif chemokine ligand 18 | 0.63 | 1.814 |
| 394 | PD-ECGF | Platelet derived endothelial cell growth factor | 0.6 | 1.26 |
| 395 | PDGF R alpha | Platelet-derived Growth Factor receptor alpha | 1.697 | 0.852 |
| 396 | PDGF R beta | Platelet-derived Growth Factor receptor beta | 0.617 | 1.464 |
| 397 | PDGF-AA | Platelet-derived growth factor AA | 0.428 | 0.667 |
| 398 | PDGF-AB | Platelet-derived growth factor AB | 0.601 | 0.919 |
| 399 | PDGF-BB | Platelet-derived growth factor BB | 0.179 | 0.449 |
| 400 | PDGF-C | Platelet-derived growth factor C | 0.435 | 0.771 |
| 401 | PDGF-D | Platelet-derived growth factor D | 0.373 | 0.376 |
| 402 | PECAM-1 /CD31 | Platelet And Endothelial Cell Adhesion Molecule 1 | 2.5 | 1.179 |
| 403 | Pentraxin3 / TSG-14 | Pentaxin-3 | 0.3 | 0.25 |
| 404 | Persephin | Persephin | 0.723 | 1.726 |
| 405 | PF4 / CXCL4 | Platelet Factor 4 | 0.166 | 0.436 |
| 406 | PlGF | Placental growth factor | 0.257 | 0.503 |
| 407 | PLUNC | Palate, Lung, and Nasal epithelium carcinoma associated protein | 0.534 | 1.429 |
| 408 | Pref-1 | Preadipocyte factor 1 | 0.579 | 1.178 |
| 409 | Progranulin | Progranulin | 0.391 | 1.769 |
| 410 | Prolactin | Prolactin | 1.016 | 1.628 |
| 411 | P-selectin | P-selectin | 0.679 | 1.426 |
| 412 | RAGE | Receptor for advanced glycation end-products | 0.704 | 0.983 |
| 413 | RANK / TNFRSF11A | Human Receptor Activator of NF-kB | 0.534 | 1.35 |
| 414 | RANTES | Regulated on activation, normal T cell expressed and secreted | 0.143 | 0.168 |
| 415 | RELM beta | Resistin-Like Molecule-beta | 0.566 | 1.607 |
| 416 | RELT / TNFRSF19L | Receptor Expressed in Lymphoid Tissue | 0.624 | 1.297 |
| 417 | ROBO4 | Roundabout Guidance Receptor 4 | 0.519 | 1.457 |
| 418 | S100 A8/A9 | S100 Calcium Binding Protein A8 | 0.62 | 1.604 |
| 419 | S100A10 | S100 Calcium Binding Protein A10 | 0.586 | 0.639 |
| 420 | SAA | Serum Amyloid A | 0.627 | 2.051 |
| 421 | SCF | Stem cell factor | 0.026 | 0.233 |
| 422 | SCF R /CD117 | Stem cell factor receptor | 0.257 | 0.47 |
| 423 | SDF-1 / CXCL12 | Stromal cell-derived factor 1 | 0.61 | 0.825 |
| 424 | sFRP-1 | Secreted frizzled-related protein 1 | 0.24 | 0.443 |
| 425 | sFRP-3 | Secreted frizzled-related protein 3 | 0.762 | 1.352 |
| 426 | sFRP-4 | Secreted frizzled-related protein 4 | 0.396 | 0.738 |
| 427 | sgp130 | IL-6 signal transducing protein gp130 | 0.572 | 0.933 |
| 428 | SIGIRR | Single immunoglobulin domain containing IL-1 receptor-related protein | 0.466 | 0.989 |
| 429 | Siglec-5/CD170 | Sialic Acid Binding Ig Like Lectin 5/Cluster of differentiation 170 | 0.517 | 0.716 |
| 430 | Siglec-9 | Sialic Acid Binding Ig Like Lectin 9 | 0.691 | 1.22 |
| 431 | SLPI | antileukoproteinase (abbr.ALP) | 1 | 1.179 |
| 432 | Smad 1 | Human mothers against DPP homolog 1 | 0.362 | 0.649 |
| 433 | Smad 4 | Human mothers against decapentaplegic homolog 4 | 0.678 | 1.803 |
| 434 | Smad 5 | Human mothers against DPP homolog 5 | 0.06 | 0.461 |
| 435 | Smad 7 | Human mothers against DPP homolog 7 | 0.451 | 1.258 |
| 436 | Smad 8 | Human mothers against DPP homolog 8 | 0.543 | 1.539 |
| 437 | SMDF / NRG1Isoform | Neuregulin 1 Isoform | 0.608 | 2.03 |
| 438 | Soggy-1 | Soggy1 | 0.969 | 1.619 |
| 439 | Sonic Hedgehog (Shh N-terminal) | Sonic Hedgehog | 0.744 | 1.145 |
| 440 | SPARC | Secreted protein acidic and rich in cysteine | 1.524 | 1.826 |
| 441 | Spinesin | Spinesin | 0.561 | 1.394 |
| 442 | TACI / TNFRSF13B | Human Transmembrane Activator and CAML Interactor | 0.567 | 1.28 |
| 443 | Tarc | Thymus and Activation-Regulated Chemokine | 0.64 | 1.085 |
| 444 | TCCR / WSX-1 | T-Cell Cytokine Receptor | 0.142 | 0.356 |
| 445 | TECK / CCL25 | Thymus-expressed Chemokine | 0.667 | 2.024 |
| 446 | TFPI | Tissue factor pathway inhibitor | 1 | 1.179 |
| 447 | TGF-alpha | Transforming growth factor alpha | 0.447 | 0.829 |
| 448 | TGF-beta 1 | Transforming growth factor-beta-1 | 0.653 | 1.779 |
| 449 | TGF-beta 2 | Transforming growth factor-beta-2 | 0.443 | 1.064 |
| 450 | TGF-beta 3 | Transforming growth factor-beta-3 | 0.03 | 0.791 |
| 451 | TGF-beta 5 | Transforming growth factor-beta-5 | 0.585 | 2.057 |
| 452 | TGF-beta RI / ALK-5 | Transforming growth factor beta receptor Ⅰ | 0.444 | 0.752 |
| 453 | TGF-beta RII | Transforming growth factor beta receptor Ⅱ | 0.683 | 1.164 |
| 454 | TGF-beta RIIb | Transforming growth factor beta receptor Ⅲ beta | 0.701 | 0.998 |
| 455 | TGF-beta RIII | Transforming growth factor beta receptor type III | 0.524 | 0.651 |
| 456 | Thrombopoietin (TPO) | Thrombopoietin | 0.758 | 0.922 |
| 457 | Thrombospondin (TSP) | Thrombospondin | 0.697 | 1.764 |
| 458 | Thrombospondin-1 | Thrombospondin-1 | 1.242 | 1.383 |
| 459 | Thrombospondin-2 | Thrombospondin-2 | 0.65 | 1.854 |
| 460 | Thrombospondin-4 | Thrombospondin-4 | 0.515 | 1.405 |
| 461 | Thymopoietin | Thymopoietin | 0.5 | 2.325 |
| 462 | Tie-1 | Tyrosine kinase with immunoglobulin and EGF homology domains-1 | 0.461 | 0.777 |
| 463 | Tie-2 | TEK tyrosine kinase | 0.308 | 0.42 |
| 464 | TIMP-1 | Tissue inhibitor of metalloproteinases-1 | 0.207 | 1.998 |
| 465 | TIMP-2 | Tissue inhibitor of metalloproteinases-2 | 0.071 | 0.53 |
| 466 | TIMP-3 | Tissue inhibitor of metalloproteinases-3 | 0.642 | 1.63 |
| 467 | TIMP-4 | Tissue inhibitor of metalloproteinases-4 | 0.185 | 0.603 |
| 468 | TL1A / TNFSF15 | TNF-like ligand 1A / TNF superfamily member 15 | 0.407 | 1.466 |
| 469 | TLR1 | Toll-like receptor 1 | 0.585 | 1.383 |
| 470 | TLR2 | Toll-like receptor 2 | 0.457 | 0.617 |
| 471 | TLR3 | Toll-like receptor 3 | 0.681 | 1.602 |
| 472 | TLR4 | Toll-like receptor 4 | 0.754 | 1.469 |
| 473 | TMEFF1 / Tomoregulin-1 | Tomoregulin 1 | 0.684 | 1.809 |
| 474 | TMEFF2 | Tomoregulin 2 | 0.313 | 0.829 |
| 475 | TNF RI / TNFRSF1A | Tumor necrosis factor receptor I | 0.52 | 0.841 |
| 476 | TNF RII / TNFRSF1B | Tumor necrosis factor receptor II | 0.657 | 1.262 |
| 477 | TNF-alpha | Tumor necrosis factor-alpha | 0.731 | 1.653 |
| 478 | TNF-beta | Tumor necrosis factor-beta | 0.537 | 1.52 |
| 479 | TRADD | Tumor necrosis factor receptor type1 associated death domain | 0.581 | 2.208 |
| 480 | TRAIL R1 / DR4 / TNFRSF10A | TNF-related apoptosis-inducing ligand receptor 1 | 0.621 | 1.93 |
| 481 | TRAIL R2 / DR5 / TNFRSF10B | TNF-related apoptosis-inducing ligand receptor 2 | 0.624 | 1.671 |
| 482 | TRAIL R3 / TNFRSF10C | TNF-related apoptosis-inducing ligand receptor 3 | 0.988 | 1.666 |
| 483 | TRAIL R4 / TNFRSF10D | TNF-related apoptosis-inducing ligand receptor 4 | 0.763 | 0.741 |
| 484 | TRAIL / TNFSF10 | TNF-related apoptosis-inducing ligand | 0.121 | 0.699 |
| 485 | TRANCE | TNF-related activation-induced cytokine | 0.401 | 0.949 |
| 486 | TREM-1 | Triggering receptor expressed on myeloid cells | 0.804 | 1.72 |
| 487 | TROY / TNFRSF19 | Tumor necrosis factor receptor superfamily, member 19 | 1.104 | 2.706 |
| 488 | TSG-6 | TNF-stimulated gene 6 protein | 0.621 | 1.992 |
| 489 | TSLP R | Thymic Stromal Lymphopoietin receptor | 0.432 | 0.992 |
| 490 | TWEAK / TNFSF12 | TNF-related and WEAK inducer of apoptosis | 0.387 | 1.123 |
| 491 | TWEAK R / TNFRSF12 | Tumor necrosis factor related Weak inducer of Apoptosis Receptor | 0.2 | 0.42 |
| 492 | Ubiquitin 1 | Ubiquitin 1 | 0.547 | 1.976 |
| 493 | uPA | Urokinase-type plasminogen activator | 0.574 | 1.626 |
| 494 | uPAR | Urokinase-type plasminogen activator receptor | 0.532 | 1.892 |
| 495 | Vasorin | Vasorin | 0.576 | 2.095 |
| 496 | VCAM-1 (CD106) | Vascular cell adhesion molecule 1 | 0.564 | 0.944 |
| 497 | VE-Cadherin | Vascular endothelial cadherin | 0.609 | 1.952 |
| 498 | VEGF | Vascular endothelial growth factor | 0.371 | 1.623 |
| 499 | VEGF R2 (KDR) | Vascular endothelial growth factor receptor2 | 0.488 | 0.572 |
| 500 | VEGF R3 | Vascular endothelial growth factor receptor3 | 0.317 | 0.419 |
| 501 | VEGF-B | Vascular endothelial growth factor B | 1.275 | 2.819 |
| 502 | VEGF-C | Vascular endothelial growth factor C | 0.754 | 1.583 |
| 503 | VEGF-D | Vascular Endothelial Growth Factor D | 0.71 | 0.941 |
| 504 | VEGI / TNFSF15 | Vascular endothelial growth inhibitor | 0.555 | 0.625 |
| 505 | WIF-1 | Wnt Inhibitory Factor-1 | 0.607 | 1.492 |
| 506 | WISP-1 / CCN4 | Wnt Inducible Signaling Pathway Protein 1 | 0.269 | 0.326 |
| 507 | XEDAR | X-linked ectodysplasin-A2 receptor | 0.615 | 0.346 |
