# Supplementary material for: Migration Inhibitory Factor in Conditioned Medium from Human Umbilical Cord Blood-Derived Mesenchymal Stromal Cells Stimulates Hair Growth
Source: Cells. 2020 May 28;9(6):1344. doi: 10.3390/cells9061344 (PMC7349163; doi:10.3390/cells9061344)
Supplement: Supplementary file 1 [file cells-09-01344-s001.zip › Supplemtal data - Skin Primary Irritation Test in humans (DSA-HSPIT001(18)-160318).pdf]

DERMAPRO SKIN RESEARCH CENTER

Skin Primary Irritation Test in Humans

48 hours Patch Test

DSA-HSPIT001(18)-16031/8

Aug. 24, 2016

FOR

MEDIPOST Co., Ltd

21, Daewangpangyo-ro 644beon-gil, Bundang-gu,
Seongnam-si, Gyeonggi-do

BY

DERMAPRO LTD.

Dermapro Skin Research Center

30 Bangbaejoongang-ro, Seocho-gu, Seoul, Korea 137-843

Final Report

Skin Primary Irritation Test in Humans

Four materials

Investigator — Eun Ju Lee

Principal Investigator — Min Kyung Shin, M.D., Ph.D.

President — Jae Sook Koh, Ph.D.

Quality Assurance Statement

This study was conducted in accordance with the intent and purpose of Good Clinical Practice (GCP) regulations that originated in the Declaration of Helsinki, as appropriate. This study report has been reviewed to assure that it correctly describes the MFDS, PCPC guideline or Standard Operating Procedure (SOP) of DERMAPRO.

- ☐ Test title : Skin Primary Irritation Test in humans
- ☐ Test code : DSA-HSPIT001(18)-16031/8

Item	Date	Confirmation of Principal investigator
Study protocol	2016.06.23	2016.06.23
Delivery product	2016.07.06	2016.07.06
Confirmation of product information	2016.07.08	2016.07.08
Recruitment	2016.07.04 ~ 07.08	2016.07.08
Test period	2016.07.12 ~ 07.15	2016.07.15
Draft report	2016.07.29	2016.07.29
Final report	2016.08.24	2016.08.24

Young Kyoung Seo
Quality Assurance

Aug. 24. 2016
Date

Jae Sook Koh, Ph.D.
President

Table of Contents

REPORT SUMMARY -- 5
1. STUDY COORDINATOR -- 6
2. INVESTIGATIVE PERSONNEL -- 6
3. CLINICAL RESEARCH STANDARD -- 6
4. PROTOCOL --- 6
5. SUBJECT -- 6
6. STUDY SCHEDULE --- 8
7. TEST MATERIAL -- 8
8. PROCEDURE -- 8
8.1. Readings and interpretation --- 9
9. DATA ANALYSIS -- 10
10. RESULT -- 10
10.1. Skin characteristics of subjects --- 10
10.2. Results -- 12
11. CONCLUSION --- 12
12. REFERENCE -- 13

APPENDIXES
APPENDIX I / SKIN CHARACTERISTICS OF SUBJECTS
APPENDIX II / RESULT OF HUMAN SKIN PRIMARY IRRITATION TEST
APPENDIX III / RESEARCH MEMBERS AND FACILITIES

REPORT SUMMARY

Title	Skin Primary Irritation Test in Humans		
Purpose	To evaluate the irritation potential of the four materials on human skin		
Test center	DERMAPRO LTD.	Test period	Jul. 12, 2016~ Jul. 15, 2016
Report date	Aug. 24, 2016		
Safety Assessor	Min Kyung Shin, M.D., Ph.D.<br>Scientific Director of Dermapro Skin Research Center		
Authentication of Test Center	DERMAPRO was validated by receiving a "QUALITY MANAGEMENT SYSTEM CERTIFICATE" (KS Q ISO 9001:2009 / ISO 9001:2008;C*ertificate No.5855*) from the KTR Certification Center for providing contract research and consulting services on human skin safety and efficacy.		
Sponsor & Supervisor	MEDIPOST Co.,Ltd<br>21, Daewangpangyo-ro 644beon-gil, Bundang-gu, Seongnam-si, Gyeonggi-do		
Methods	*Subjects*: 30 healthy women (mean age 43.2 ± 6.9 yrs)<br>*Procedure*: 48 hrs single closed patch test on the upper back.<br>*Reading and interpretation*: Reactions were assessed at 30 mins and 24 hrs after patch removal by the dermatologist and principal researcher according to the modification of Frosch & Kligman (1979). All assessments were performed under standard lighting conditions.		
Results	Among the four materials, one (#1) showed skin reaction of 1+ grade in a subject and the others did not induce any skin reaction in all subjects during the course of study.		
Conclusions	In respect of the primary irritation potential on human skin, these four materials can be considered that within the scope of the slight-irritation.		
Appendix	Skin characteristic of subjects, Result, Research members and facilities		

1. STUDY COORDINATOR

Monitor: Eun Su Jeon

Sponsor: MEDIPOST Co.,Ltd

2. INVESTIGATIVE PERSONNEL

Investigator: Eun Ju Lee

Principal Investigator: Min Kyung Shin, M.D., Ph.D.

3. CLINICAL RESEARCH STANDARD

This study was conducted according to applicable Good Clinical Practices (GCP) and the Standard Operating Procedures (SOPs) of Dermapro Skin Research Center.

4. PROTOCOL

The study protocol was followed with the consent agreement, subject selection and skin characteristics of subject attached in Appendix I.

5. SUBJECT

5.1. Principle of recruitment

The subjects must be clearly informed, verbally and in writing, regarding the nature of the study, the timetable, possible risks and constraints. The investigator will screen the participation form of the volunteer and subject selection will be based on the fulfillment of the study's inclusion and exclusion criteria.

The study inclusion and exclusion criteria are as follows:

5.2. Inclusion criteria

1) Healthy females aged 20 to 50 years.
2) Signed and informed consent; the purpose and the protocol of the study were explained to subjects.

3) Subjects agree to follow the conditions specified in the Study Information Sheet
4) Volunteers cooperative and available during the study period.

5.3. Exclusion criteria

1) Pregnancy or nursing condition or planning to become pregnant within six months.
2) Damaged skin, in or around the test site, which includes sunburn, tattoos, scars or other disfiguration of the test site.
3) Any active skin disease which may interfere with the aim(s) of the study
4) Taking medicine for which may interfere with the aim(s) of the study
5) Allergic to cosmetics, topical drug and usual sunlight exposure
6) In case of atopic dermatitis
7) Taking contraceptive, antihistaminic and anti-inflammatory drug
8) Chronic disease (diabetes, asthma, high blood-pressure).
9) Allergic to tapes
10) Participation in another simultaneous study
11) Participation in a previous study without an appropriate intervening period (six months) between studies.
12) Any difficult which may interfere with the aim of the study as the judgment of the investigator.

5.4. Prohibition and restriction

1) Contact with water on the test site is forbidden during attaching patch.
2) Use of anti-inflammatory drugs or antihistaminic or systemic steroids, by a general route, is forbidden throughout the duration of the study.

5.5. Subject withdrawal

After admission to the study, the subject may withdrawal at any time for any reason, but must report such reason fairly and accurately.

Subjects failing to complete the study will be identified, and whenever possible, a reason will be given. Subjects who drop out of the study will not be replaced.

1) They suffer any illness or accident or develop any condition during the study which

could affect the outcome of the study.

2) They suffer of any side effect occurred by test material during the study.
3) They do not follow the conditions of the Study Information Sheet or they no longer wish to participate in the study.
4) They could be dropped off from the study by investigator when they affect the outcome of the study.

6. STUDY SCHEDULE

Start Date: Jul. 12, 2016

Finish Date: Jul. 15, 2016

7. TEST MATERIAL

The following test materials were supplied from the sponsor for this study.

Table 1. Name of test materials supplied from sponsor

No	Product Name	Lot No.	Active ingredient	Type	Conc.
1	100% CM	MPCM1602	Effective proteins for prevent hair loss (100%)	Liquid	As is
2	50% CM	MPCM1602	Effective proteins for prevent hair loss (50%)	Liquid	As is
3	5% Essence	-	-	Essence	As is
4	Placebo	-	-	Essence	As is
5	Negative control	-	-	Liquid	As is

8. PROCEDURE

Patch test was performed in healthy subjects who had no history of allergenic contact dermatitis. The subjects sit in a relaxed normal position, the back slightly bent forwards. The test materials were applied to healthy skin on the back, which is to be free of ointments and excessive sebum. It is recommended that subjects take a shower or bath in the morning before testing. If necessary, the skin can be cleaned by alcohol. Sixteen microliters of the material was applied on a filter paper in chamber (Finn Chambers® on Scanpor, SmartPractice, Denmark) and fixed to the skin with adhesive tape. The

preferred test site was the upper back and the subjects should be informed about avoiding excessive exercise, showers, etc. to keep the test system dry.

The patch test system was usually removed after 48 hours, as modified by Frosch & Kligman (1979)[1)], and readings were done 30 minutes after removal of the strips (day 2) and after 24 (day 3) or 48 (day 4) hours. All assessments were performed under standard lighting conditions by the responsible expert researcher or dermatologist. Subjects were instructed to keep the test sites dry over the application period.

8.1. Readings and interpretation

Readings made at day 2 and 3 and reactions were graded according to the modification of Frosch & Kligman (1979)[1)].

Patch test reactions are classified and recorded as follows (Table 2).

Table 2. Clinical standard photographs of visual assessment for human patch test

Description	Slight erythema, either spotty or diffuse	Moderate uniform erythema	Intense erythema with edema	Intense erythema with edema & vesicle
After 30 min	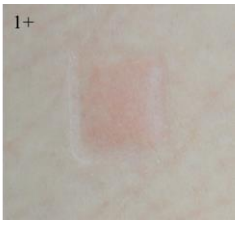	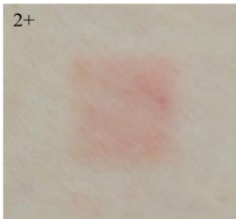	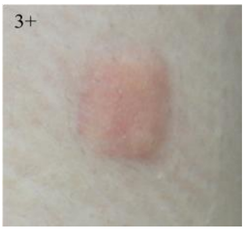	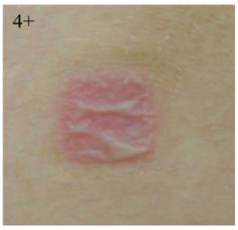
After 24 hr	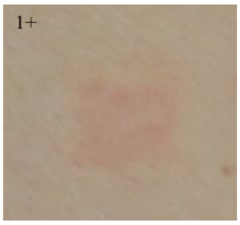	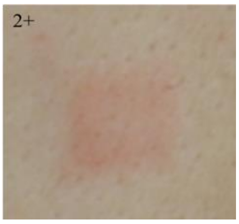	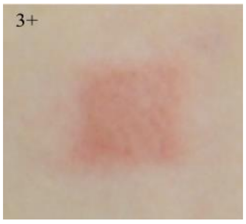	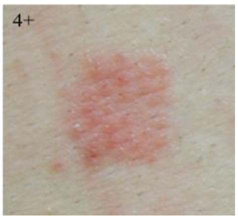

9. DATA ANALYSIS

Reaction grade were derived by application of the following formulae. And the result was judged by the criteria in Table 3.

$$\text{Response} = \frac{\sum (\text{Grade} \times \text{No. of Responders})}{4\ (\text{Maximum grade}) \times n\ (\text{Total Subjects})} \times 100 \times 1/2$$

Table 3. Human primary irritation index for cosmetic products[11)]

Range of Response	Criteria
0.00 ≤ R < 0.87	Slight
0.87 ≤ R < 2.42	Mild
2.42 ≤ R < 3.44	Moderate
3.44 ≤ R	Severe

10. RESULT

10.1.Skin characteristics of subjects

For conducting this study, 30 subjects were recruited. The data of 30 subjects (average 43.2±6.9yrs) was collected (Appendix I). The physical characteristics of the study subjects were summarized in Table 4.

Table 4. Skin characteristics of subjects (n=30)

Item	Classification	Frequency (n)	Percentage (%)
Skin Type	Dry skin	5	16.67
	Normal skin	10	33.33
	Oily skin	2	6.67
	Dry to oily skin	13	43.33
	Problem skin	0	0.00
Irritability	Yes	0	0.00

	No	30	100.00
Stinging	Yes	0	0.00
	No	30	100.00
Side effects by cosmetics	Yes	0	0.00
	No	30	100.00
Allergy*	Food allergy	0	0.00
	Metal allergy	2	6.67
	Photoallergy	0	0.00
	Extra allergy	0	0.00
	No	28	93.33
Skin diseases	Acne	0	0.00
	Atopy	0	0.00
	Hair loss	0	0.00
	Extra skin diseases	0	0.00
	No	30	100.00
Tight feeling	Yes	0	0.00
	No	30	100.00
Taking supplements	Taking korean herb medicines	0	0.00
	Taking nutrients	7	23.33
	Taking extra supplements	0	0.00
	No	23	76.67
Smoking	No	29	96.67
	Less than 10 pieces	1	3.33
	More than 10 pieces	0	0.00
Menstrual cycle	During menstruation	5	16.67
	Within 1 week before menstruation	3	10.00
	Within 1 week after menstruation	8	26.67
	The others	14	46.67

*Metal allergy did not affect the test results.

10.2. Results

Data table for the grades of the skin reactions at 30 minutes and 24 hours after patch removal is found in Appendix II.

Among the four materials, one (#1) showed skin reaction of 1+ grade in a subject and the others did not induce any skin reaction in all subjects during the course of study.
The score of the skin reaction is as follows:

Table 5. Results of skin primary irritation test on humans (n=30)

No	Test material	No. of Responder	48 hrs				72 hrs				Reaction Grade		
			1+	2+	3+	4+	1+	2+	3+	4+	48h	72h	Mean
1	100% CM	1	1	-	-	-	-	-	-	-	0.8	0.0	0.4
2	50% CM	0	-	-	-	-	-	-	-	-	0.0	0.0	0.0
3	5% Essence	0	-	-	-	-	-	-	-	-	0.0	0.0	0.0
4	Placebo	0	-	-	-	-	-	-	-	-	0.0	0.0	0.0
5	Negative control	0	-	-	-	-	-	-	-	-	0.0	0.0	0.0

11. CONCLUSION

In respect of the primary irritation potential on human skin, these four materials can be considered that within the scope of the slight-irritation.

12. REFERENCE

1) Frosch PJ, KligmanAM. The soap chamber test. A new method for assessing the irritancy of soaps. J Am Acad Dermatol. 1979 1(1): 35-41.
2) Fisher T, Maibach HI. Finn chamber patch test technique. Contact dermatitis. 1984 11(3): 137-40.
3) Francis NM, Maibach HI. Dermatotoxicology (4thed). Hemisphere Publishing Corporation, New York. 1991 p143-452.
4) James GM, Vincent AD. Patch testing. Contact and occupational dermatology. St. Louis: Mosby Year Book. 1992 p 30-49.
5) Dooms-Goossens A. Patch testing without a kit. 1995 In:Guyin JD (ed) Practical contact dermatitis. McGraw-Hill, New York, 63-74.
6) Walker AP, Basketter DA, Baverel M, Diembeck W, Matties W, Mougin D, Paye M, Rothlisverger R, Dupuis J. Test guidelines for assessment of skin compatibility of cosmetic finished products in man. Task Force of COLIPA. Food Chem Toxicol. 1996 34(7): 651-60.
7) Basketter DA, Chamnerlain M, Griffiths HA, Rowson M, Whittle E, York M. The classification of skin irritants by human patch test. Food ChemToxicol. 1997 35(8): 845-52.
8) Bourke J, Coulson I, English J. Guidelines for care of contact dermatitis. Br J Dermatol. 2001 145(6): 877-85.
9) Devos SA, Van Der Valk PG. Epicutaneous patch testing. Eur J Dermatol. 2002 12(5): 506-13.
10) Kim E, Maibach HI. Changing paradigms in dermatology: science and art of diagnostic patch and contact urticaria testing. ClinDermatol. 2003 21(5): 346-52.
11) An SM, Ham H, Choi EJ, Shin MK, An SS, Kim HO, Koh JS. Primary irritation index and safety zone of cosmetics: Retrospective analysis of skin patch tests in 7,440 Korean women during 12 years. Int J Cosmet Sci. 2014 36(1): 62-67.
12) Loretz LJ. Safety Evaluation Guidelines. Personal Care Products Council 2014

APPENDIXES

APPENDIX I

SKIN CHARACTERISTIC OF SUBJECTS

Appendix I. Skin characteristics of subjects

No.	Name	Age	Sex	Skin type	Irritability	Stinging sensation	Side effects of cosmetics	Allergy	Skin diseases	Tight feeling	Taking supplem-ents	Smoking	Menstrual cycle
1	H*M	44	F	4	2	2	2	5	5	2	4	1	3
2	K*H	47	F	4	2	2	2	5	5	2	4	2	4
3	P*O	46	F	2	2	2	2	5	5	2	4	1	1
4	K*J	43	F	1	2	2	2	5	5	2	4	1	3
5	K*Y	44	F	2	2	2	2	5	5	2	2	1	2
6	C*Y	40	F	3	2	2	2	5	5	2	4	1	1
7	P*A	44	F	4	2	2	2	5	5	2	2	1	4
8	P*J	28	F	4	2	2	2	5	5	2	4	1	4
9	J*H	45	F	2	2	2	2	5	5	2	4	1	1
11	N*S	42	F	4	2	2	2	5	5	2	4	1	2
12	H*H	48	F	2	2	2	2	5	5	2	4	1	4
13	Y*S	25	F	4	2	2	2	2	5	2	4	1	4
14	J*N	49	F	1	2	2	2	5	5	2	4	1	1
15	P*H	48	F	2	2	2	2	5	5	2	2	1	3
16	S*R	43	F	4	2	2	2	5	5	2	4	1	3
17	L*S	46	F	2	2	2	2	5	5	2	2	1	4
18	L*J	45	F	4	2	2	2	5	5	2	4	1	4
19	L*J	45	F	2	2	2	2	5	5	2	4	1	3
20	K*M	48	F	4	2	2	2	5	5	2	4	1	4
21	K*S	47	F	2	2	2	2	2	5	2	2	1	4
22	K*S	40	F	3	2	2	2	5	5	2	4	1	2
23	K*S	47	F	1	2	2	2	5	5	2	4	1	3
24	L*S	50	F	1	2	2	2	5	5	2	4	1	4
25	K*J	44	F	4	2	2	2	5	5	2	4	1	1
26	P*Y	49	F	4	2	2	2	5	5	2	2	1	4
27	K*K	48	F	4	2	2	2	5	5	2	4	1	4
28	K*J	49	F	2	2	2	2	5	5	2	4	1	4
29	C*J	34	F	2	2	2	2	5	5	2	2	1	3
30	P*Y	44	F	4	2	2	2	5	5	2	4	1	3
31	C*S	23	F	1	2	2	2	5	5	2	4	1	4
Total Subject Number = 30													

1)Skin type: dry(1), nomal(2), oily (3), dry to oily(4), problematic skin(5)**, 2)Irritability:** yes(1), no(2) **3)Stinging sensation:** yes(1), no(2), **4)Side effects of cosmetics:** yes(1), no(2), **5)Allergy:** food(1), metal(2) photoallergy(3), extra(4), none(5), **6)Skin disease**: acne(1), atopy;(2), hair loss(3), extra(4), none(5), **7)Tight feeling:** yes(1), no(2), **8)Taking supplements :** taking korean herb medicines(1),taking nutrients(2), extra(3), none(4), **9)Smoking(day):** no(1), less than 10 pieces(2), more than 10 pieces(3), **10)Menstrual cycle :** within 1 week before menstruation(1), during menstruation(2), within 1 week after menstruation(3), the others(4)

APPENDIX II

RESULT

Appendix II. Result sheet

No.	Age	Hr	**Test materials**				
			1	2	3	4	5
1	44	48					
		72					
2	47	48					
		72					
3	46	48					
		72					
4	43	48					
		72					
5	44	48					
		72					
6	40	48					
		72					
7	44	48					
		72					
8	28	48					
		72					
9	45	48					
		72					
11	42	48					
		72					
12	48	48					
		72					
13	25	48					
		72					
14	49	48					
		72					
15	48	48					
		72					
16	43	48					
		72					
17	46	48					
		72					
18	45	48					
		72					
19	45	48					
		72					
20	48	48					
		72					
21	47	48					
		72					
22	40	48					
		72					
23	47	48					
		72					
24	50	48					
		72					
25	44	48					
		72					
26	49	48	+				
		72					
27	48	48					
		72					
28	49	48					
		72					
29	34	48					
		72					
30	44	48					
		72					
31	23	48					
		72					

APPENDIX III

RESEARCH MEMBERS & FACILITIES

APPENDIX III

RESEARCH MEMBERS & FACILITIES

1. COMPANY INFORMATION

Company name	DERMAPRO LTD.
Business fields	Human clinical evaluation of efficacy and safety for cosmetics, cosmeceuticals, toiletries and neutraceuticals
Head office	30, Bangbaejoongang-ro, Seocho-gu, Seoul, Korea 137-843
CEO	Jae Sook Koh, Ph.D.
Scientific Director	Jae Sook Koh, Ph.D.
	Min Kyung Shin, M.D., Ph.D.
	Sang Jun Lee, M.D., Ph.D.

2. CLINICAL EVALUATION FIELDS

Classification	Test Item
Efficacy Evaluation for MFDS (I)	Human Skin Wrinkle Test (I)
	Human Skin Lightening Effect Test (I)
	Sun protection of UVB, UVA Test
	Water Proof SPF Test
	Hair Loss Prevention and Growth Effect Test (I)
	Human Skin Wrinkle Test of Food Supplement
	Human Skin Lightening Effect Test of Food Supplement
	Human Skin Hydration Test of Food Supplement
	Acne Skin Improvement Effect Test
Efficacy Evaluation (II)	Human Skin Wrinkle Test (II)
	Human Skin Lightening Effect Test (II)
	Sweat Resistance SPF Test
	Human Skin Firming & Sagging Test
	Body Slimming Effect Test
	Human Skin Water Holding Capacity Test of 24 Hours

	Human Skin Scale Test
	Human Skin Radiance & Gloss Test
	Damaged Skin Recovery Effect Test (I), (II), (III)
	Cleansing Effect Test
Body Evaluation	Human Breast Firming Test
	Cellulite Reduction Test
Make-Up Evaluation	Evaluation of Lip Products
	Cleansing Effect Test
	Evaluation of Make-up
	Evaluation of Mascara
Hair & Scale Evaluation	Hair Loss Prevention and Growth Effect Test (II)
	Evaluation of Hair products
	Eyelash Growth Effect Test
	Hair Dandruff and Itching Improvement Effect Test
	Human Hair *ex vivo* Test
	Scalp condition Test
Safety Evaluation	Skin Primary Irritation Test
	Skin Repeat Insult Patch Test
	Skin Sting Test
	Skin Photo-irritation Test
	Skin Cumulative Irritation Test
	Eye Tolerance Test
	Eye Rim Use Test
	Skin Adverse Reaction Test
	Skin Usage Test
	Skin Photo-Sensitization Test
Antimicrobial Efficacy Evaluation	Skin Antimicrobial Efficacy Test
Sensory Evaluation	Sensory Test

Food Supplement	Human Skin Wrinkle Test of Food Supplement
	Human Skin Lightening Effect Test of Food Supplement
	Human Skin Hydration Test of Food Supplement
in vitro Assay	Skin Lightening Effect Test
	Anti-wrinkle Effect Test
	Antioxidant Activity Test
	Skin Absorption Test
	in vitro SPF Test

3. RESEARCH TEAM

1) Chief of Research : Min Kyung Shin;Dermatologist
Graduated with a M.D., Ph.D.in Medical Science from College of Medicine, Kyung Hee University

2) Researcher : Eun Ju Lee
Certificate of course completion with a Master's degree in Immunology from Seoul National University

3) Co-researcher : Eunseok Lee
Graduated with a Associate's degree in Food Science & Bio Technology from Ansan College of Technology

4) QA team
Young Kyoung Seo : Efficacy Team, QA Team
Graduated with a Master's degree in Physiology from College of Medicine, Chung-Ang University

Ji Hwoon Baek :
Graduated with a Ph.D. in Medical Science from School of Medicine, Yonsei University

4. RESEARCH FACILITIES

Device	Description
DermaScan® C Ver.3	To visualize layers of the skin and subcutaneous tissue and to make approximate dimensional measurements
Neurometer® CPT	To measure perception and pain thresholds
Facial ion Steamer®	To Activate the hydrophilic factor and replenish water in skin
Periscan PIM II® Laser Doppler Perfusion Imaging system	To measure the circulation of microvessels
SquameScan® 850A	To measure the desquamation of the skin
Mexameter® MX 18	To measure the melanin and the erythema index of the skin
Spectrophotometer® CM-2500d	To measure the Lightness (L*), Green-Red (a*) and Blue-Yellow (b*) of the skin
Cutometer® MPA 580	To measure the viscoelasticity of the skin for face
Reviscometer®	To measure the reviscoelasticity of the skin
Sebumeter® SM 810	To measure the sebum content variation in the stratum corneum
Corneometer® CM 825	To measure the water content variation in the stratum corneum
Moisturemeter® SC	To measure the water content variation in the stratum corneum
Moisturemeter® D	To measure the water content variation in the dermis
Skin-pH-Meter® pH 900	To measure the pH variation in the stratum corneum
Tewameter® TM 300	To measure the transepidermal water loss
Vapometer®	To measure the transepidermal water loss in the stratum
Skin Visiometer® SV 600	To analyze the skin roughness
UVB Irradiator	To induce artificial pigmentation
Radiometer VLX-3W	To measure the UV A and B irradiance
Photometer/Radiometer PMA2100	Detector
One port solar simulator LS-1000	To measure the SPF & PA value

Multiport Solar UV Simulator (601-300W)	To measure the SPF & PA value
Ultraviolet Meter Model 3D-600	Detector
Sauna room system	To use for SPF/PA test (sweat resistance)
Whirlpool massage system	To use for SPF/PA test (water proof)
Inbody® 330	To measure the body mass
Cometech QC-508E	To analyze hair length force, tension, extension and pushing force
Digital still camera DSC-S75	To take a picture of the skin
Digital still camera D300	To take a picture of the skin
Digital still camera D40	To take a picture of the skin
Digital still camera D90	To take a picture of the skin
Visioscan® VC 98	To estimate the desquamation and the sebum
Facial stage® DM 3	To take a picture of face under wood lamp and fluorescent light
ThemaCam T360	To measure a temperature on the skin
Charm View®	To examine the skin surface
Moire topography	To take a picture of the skin
Digital camera Powershot A520	To take a picture of the hair
Trichogram system (Trichoscan professional ver. 2.0)	To evaluate the hair loss
Folliscope2.8®	To evaluate the hair loss
Environmental control unit (DKA-3U)	To maintain the temperature and humidity
Environmental control unit (BCTH-A075SU)	To maintain the temperature and humidity
Environmental control unit (HT-A3GG3)	To maintain the temperature and humidity
Image Analyzer (Image-pro® plus 5.1)	To analysis the image
Vivascope® 1500	To take a picture of the melanin

PRIMOS® face & body	To visualize an exact 3D image in vivo measurement of human body skin surface
PRIMOS® premium	To visualize an exact 3D image in vivo measurement of human facial skin surfaces
PRIMOS® lite	To visualize an exact 3D image in vivo measurement of human body skin surface
PRIMOS® shapescan	To visualize an exact 3D image in vivo measurement of human facial skin surfaces
AramoTS®	To take a picture of the human body skin surface
VISIA®	To take a picture of face under wood lamp and fluorescent light
Glossmeter®	To measure the gloss of the skin
DERMALAB®	To measure the water content variation
VisioLine®	To analyze the skin roughness
Radioscan	To measure the skin transparency
NeoScope JCM-5000®	To scan electron microscope
Inbody® S10	To measure body (intracellular and extracellular water) water state
ECOSKIN®	To analyze an porphyrin and photoaging value in skin surface image
SLIT LAMP® (SL-7F)	To visualize an cornea image in direct Installation test
SPOT THERMOMETER® (HT-20)	To measure the skin temperature
Universal Testing Machine	To measure the tensile load, combing force, frictional force, stiffness and bending(flexural) force of hair
Hair luster system	To evaluate the hair luster ring
UV irradiation system	To detract the hair by UVB
Hair retentivity chamber	To evaluate the formation and maintenance of hair curling
Moisture analyzer (XM-60-HR)	To measure the moisture content of hair
Epsilon	To capture dielectric permittivity image
Janus	To take a picture of face under wood lamp and fluorescent light
F-ray	To take a picture of the skin

Dermal Torque Meter DTM310	To measure the viscoelasticity of the skin
Torsional Ballistometer BLS780	To measure the viscoelasticity of the skin
Antera 3D CS	To evaluate the color on the skin
Translucency meter	To measure the translucency on the skin

5. RESEARCH ACCOMPLISHMENT

1) 4-n-butylresorcinol dissolving microneedle patch for skin depigmentation: a randomized, double-blind, placebo-controlled trial. Kim S, Yang H, Kim M, Baek JH, Kim SJ, An SM, Koh JS, Seo R, Jung H. *Journal of Cosmetic Dermatology*. 2016 in press
2) Evaluation of the Anti-wrinkle Effect of an Ascorbic Acid-loaded Dissolving Microneedle Patch via a Double-blind, Placebo-controlled Clinical Study. Lee C, Yang H, Kim S, Kim H, Kim N, An S, Koh J, Jung H. *International Journal of Cosmetic Science* 2015 in press
3) A proposal of a standardized protocol to evaluate waterproof effect of eyeliner and mascara. Kim MJ, Seo YK, Boo YC, Koh JS. *International Journal of Cosmetic Science* 2015 in press
4) Itching sensation and neuronal sensitivity of the skin. Ham H, An SM, Lee EJ, Lee E, Kim HO, Koh JS. *Skin Research and Technology* 2015 in press
5) Analysis of comedone, sebum and porphyrin on the face and body for comedogenicity assay. Baek JH, An SM, Choi KM, Jung MK, Shin MK, Koh JS. *Skin Research and Technology* 2015 in press
6) Scutellaria radix Extract as a Natural UV Protectant for Human Skin. Seok JK, Kwak JY, Choi GW, An SM, Kwak JH, Seo HH, Suh HJ, Boo YC. *Phytotherapy Research*. 2015 in press
7) Clinical evidence of effects of Lactobacillus plantarum HY7714 on skin aging: a randomized, double blind, placebo-controlled study. Lee DE, Huh CS, Ra J, Choi ID, Jeong JW, Kim SH, Ryu JH, Seo YK, Koh JS, Lee JH, Sim JH, Ahn YT. *Journal of Microbiology and Biotechnology* 2015 in press
8) 4-n-butylresorcinol dissolving microneedle patch for skin depigmentation: a randomized, double-blind, placebo-controlled trial. Kim S, Yang H, Kim M, Baek JH, Kim SJ, An SM, Koh JS, Seo R, Jung H. *Journal of Cosmetic Dermatology*. 2015 in press
9) Identification of a possible susceptibility locus for UVB-induced skin tanning phenotype in Korean females using genomewide association study. *Experimental Dermatology*. Kwak TJ, Chang YH, Shin YA, Shin JM, Kim JH, Lim SK, Lee SH, Lee MG, Yoon TJ, Kim CD, Lee JH, Koh JS, Seo YK, Chang MY, Lee Y. 2015 in press
10) The Atopic Dermatitis Antecubital Severity score: validity, reliability, and sensitivity to change in patients with atopic dermatitis. *International Journal of Dermatology* Baek JH, Park CW, Choi KM, Yang YS, Lee SY, Koh JS, Chung BY, Kim HO, Park GH. 54(12):1382-9. Dec. 2015
11) A study of the human skin-whitening

effects of resveratryl triacetate. Ryu JH, Seok JK, An SM, Baek JH, Koh JS, Boo YC. *Archives of Dermatological Research* 307(3):239-47. Apr 2015

12) Dermal matrix affects translucency of incident light on the skin. Kim HJ, Baek JH, Eo JE, Choi KM, Shin MK, Koh JS. *Skin Research and Technology* 21(1):41-6. Feb 2015
13) The seasonal variation in skin hydration, sebum, scaliness, brightness and elasticity in Korean females. Nam GW, Baek JH, Koh JS, Hwang JK. *Skin Research and Technology* 21(1):1-8. Feb. 2015
14) Pore volume is most highly correlated with the visual assessment of skin pores. Kim SJ, Shin MK, Back JH, Koh JS. *Skin Research and Technology* 20(4):429-34. Nov 2014.
15) The quantitative analysis of spreadability, coverage, and adhesion effect after application of the base make-up product. Lee SY, Baek JH, Shin MK, Koh JS. *Skin Research and Technology* 20(3):341-6. Aug 2014.
16) Effects of resveratrol, oxyresveratrol, and their acetylated derivatives on cellular melanogenesis. *Archives of Dermatological Research* Park J, Park JH, Suh HJ, Lee IC, Koh J, Boo YC. 306(5):475-87 Jul 2014
17) Bioengineering methods and instrumentation of the skin color. Baek JH, Shin MK, Koh JS. *Korean Journal of Cosmetic Dermatology* 11:9-13. June 2014
18) A quantitative evaluation method of skin texture affected by skin ageing using replica images of the cheek. Ryu JH, Seo YK, Boo YC, Chang MY, Kwak TJ, Koh JS. *International Journal of Cosmetic Science* 36(3):247-52. Jun 2014
19) Screening for anti-inflammatory activities in extracts from Korean herb medicines. An SM, Kim HG, Choi EJ, Hwang HH, Lee E, Baek JH, Boo YC, Koh JS. *Journal of the Society of Cosmetic Scientists of Korea* 40(1):95-10. Mar 2014
20) Primary irritation index and safety zone of cosmetics: retrospective analysis of skin patch tests in 7440 Korean women during 12 years. An SM, Ham H, Choi EJ, Shin MK, An SS, Kim HO and Koh JS. *International Journal of Cosmetic Science* 36(1): 62-67. Feb. 2014
21) Protective effects of skin permeable epidermal and fibroblast growth factor against ultraviolet-induced skin damage and human skin wrinkles. An JJ, Eum WS, Kwon HS, Koh JS, Lee SY, Baek JH, Cho YJ, Kim DW, Han KH, Park J, Jang SH, Choi SY. *Journal of Cosmetic Dermatology*. 12(4):287-95. Dec. 2013
22) A validation study to find highly correlated parameters with visual assessment for clinical evaluation of

cosmetic anti-cellulite products. Yoo MA, Seo YK, Ryu JH, Back JH, Koh JS. *Skin Research and Technology* 0:1-8. July 2013

23) Characterization of microthermal zones induced by fractional radiofrequency using reflectance confocal microscopy: A preliminary study. Shin MK, Park JM, Lim HK, Choi JH, Baek JH, Kim HJ, Koh JS, Lee MH. *Lasers in Surgery and Medicine*. 45(9):503-8. Oct. 2013.

24) 기능성 화장품의 인체시험 설계 및 통계적용 방법에 대한 고찰. 서영경, 고재숙, 이원철. *대한화장품학회지* 39(2):105-115. Jun. 2013.

25) Characteristic aging features in Korean women's hair and scalp. Kim SN, Lee SY, Choi MH, Joo KM, Kim SH, Koh JS, Park WS. *The British Journal of Dermatology*. 168(6):1215-23. Jun. 2013

26) Acute Modulations in Stratum Corneum Permeability Barrier Function Affect Claudin Expression and Epidermal Tight Junction Function via Changes of Epidermal Calcium Gradient. Baek JH, Lee SE, Choi KJ, Choi EH, Lee SH. *Yonsei Medical Journal*. 1;54(2):523-8. Mar. 2013.

27) Analysis of the temporal change in biophysical parameters after fractional laser treatments using reflectance confocal microscopy. Shin MK, Kim MJ, Baek JH, Yoo MA, Koh JS, Lee SJ, Lee MH. *Skin Research and Technology* 19:e515–e520. Feb. 2013.

28) Assessment of the Breast-Firmming Effects of a Cosmetic Preparation with Moiré Tophography in Combination with 2D and 3D Digital Image Analyses. Seo YK, Yoo MA, Ryu JH, Kim SJ, Cho SA, Nam GW, Cho JC, Boo YC, Koh JS. *Journal of the Society of Cosmetic Scientists of Korea* 38(4):289-296. Dec. 2012

29) Complementary and Alternative Medicine - Botanical Cosmeceuticals. Chapter 2. Safety testing of cosmetics. Koh JS. *Transworld Research Network* 23-47. 2011

30) Instrumental Assessments of Sub-clinical Skin Reactions induced by Cosmetic Ingredients. An SM, Lee MY, Baek JH, Ham H, Boo YC, Koh JS. *Journal of the Society of Cosmetic Scientists of Korea*. 38(1):43-50. Mar. 2012.

31) Evaluation of the clinical efficacy of fractional radiofrequency microneedle treatment in acne scars and large facial pores. Cho SI, Chung BY, Choi MG, Baek JH, Cho HJ, Park CW, Lee CH, Kim HO. *Dermatologic Surgery*. 38(7 Pt 1):1017-1024. Jul. 2012

32) Screening of plant extracts for human tyrosinase inhibiting effects. Kim M, Park J, Song K, Kim HG, Koh JS, Boo YC. *International Journal of Cosmetic Science*. 34(2):202-8. Apr. 2012.

33) Reduction of facial wrinkles depth by sleeping on copper oxide-containing pillowcases: a double blind, placebo controlled, parallel, randomized. Baek JH, Yoo MA, Koh JS, Borkow G. *Journal of Cosmetic Dermatology* 11:1-8. Dec. 2011
34) Effects of a new mild shampoo for preventing hair loss in Asian by a simple hand-held phototrichogram technique. Baek JH, Lee SY, Yoo M, Park WS, Lee SJ, Boo YC, Koh JS. *International Journal of Cosmetic Science*. 33(6):491-496. Dec. 2011
35) Cosmetic efficacy evaluation of an anti-acne cream using the 3D image analysis system. Choi KM, Kim SJ, Baek JH, Kang SJ, Boo YC, Koh JS. *Skin Research and Technology*. 18(2):192-8. Sep. 2011
36) Comparison of the antimelanogenic effects of *p*-coumaric acid and its methyl ester and their skin permeabilities. Song K, An SM, Kim M, Koh JS, Boo YC. *Journal of Dermatological Science*. 63(1):17-22. Jul. 2011
37) Relationship between clinical features of facial dry skin and biophysical parameters in Asians. Baek JH, Lee MY, Koh JS. *International Journal of Cosmetic Science*. 33(3):222-227. Jun. 2011
38) 큰뱀무 추출물의 항산화 활성 및 항노화에 관한 연구. 김민지, 양현갑, 박수남. *대한화장품학회지*. 37(2):191-198. Jun. 2011
39) Effects of *p*-coumaric acid on erythema and pigmentation of human skin exposed to ultraviolet radiation. Seo YK, Kim SJ, Boo YC, Baek JH, Lee SH, Koh JS. *Clinical and Experimental Dermatology*. 36(3):260-266. Apr. 2011
40) Use of non-melanocytic HEK293 cells stably expressing human tyrosinase for the screening of anti-melanogenic agents. Kim M, An SM, Koh JS, Jang DI, Boo YC. *Journal of cosmetic science*. 62(5):515-23. Sep-Oct. 2011
41) Retinyl retinoate, a novel hybrid vitamin derivative, improves photoaged skin: a double-blind, randomized-controlled trial. Kim H, Koh J, Baek J, Seo Y, Kim B, Kim J, Lee J, Ryoo H, Jung H. *Skin Research and technology*. 17(3):380-385. Aug. 2011
42) 양파껍질 추출물의 항균, 항산화 및 항노화 효과에 관한 연구. 김정은, 김아름, 김민지, 박수남. *공업화학회지*. 22(2):178-184. Apr. 2011
43) Effects of plant extract-containing creams on UVB radiation-induced inflammatory responses in mice. An SM, Lee SJ, Koh J-S, Park KM, Boo YC. *Journal of the Society of Cosmetic Scientists of Korea*. 36(4):271-280. 2010
44) *p*-Coumaric acid not only inhibits human tyrosinase activity in vitro but also melanogenesis in cells exposed to UVB.

An SM, Koh J-S, Boo YC. *Phytotherqpy Research*, 24:1175–1180. 2010

45) 잣나무 잎 추출물의 항균 및 항산화 활성과 성분분석. 김정은, 김우연, 김지욱, 박현수, 이승훈, 이순영, 김민지, 김아름, 박수남. *대한화장품 학회지*. 35(4):303-314. Dec. 2010

46) *Quercus glauca* extract and rutin inhibit the UVB-induced expression of matrix metalloproteinase-1 in human dermal fibroblasts. Lee SJ, Koh J-S, Ha B-J, Boo YC. *Journal of the Korean Society for Applied Biological Chemistry*. 53(6):677-684. 2010

47) Inhibitory Effects of Neo Muscat Grape Vine Extracts on Melanin Biosynthesis. Choi SY, Kong YH, Lee Y, Baek JH, Lee SH, Lee P. *Journal of Applied Biological Chemistry*. 53(5):566-569. 2010

48) *Acanthopanax koreanum* Fruit Waste Inhibits Lipopolysaccharide-Induced Production of Nitric Oxide and Prostaglandin E_2 in RAW264.7Macrophages. Yang EJ, Moon JY, Lee JS, Koh JS, Lee NH, Hyun CG. *Journal of Biomedicine and Biotechnology*. doi: 10.1155/2010/715739. Mar. 2010

49) Inhibition of melanogenesis by tyrosinase siRNA in human melanocytes. An SM, Koh J-S Boo YC. *BMB reports*. 42(3):178-183, 2009

50) Electrogenerated Chemiluminescence Sensor Based on Tris(2,2'-bipyridyl) ruthenium(II) Immobilized in the Composite Film of Multi-walled Carbon Nanotube/Sol-gel Zinc oxide/Nafion. Choi EJ, Kang CH, Choi HN, Lee WY. *Bulletin of the Korean Chemical Society*. 30(10):2387-2392. Sep. 2009

51) Evidence for the association of peroxidases with the antioxidant effect of *p*-coumaric acid in endothelial cells exposed to high glucose plus arachidonic acid. Lee SJ, Mun GI, An SM, Boo YC. *BMB Reports*. 42(9):561-567. Sep. 2009

52) Differential gene expression in young and senescent endothelial cells under static and laminar shear stress conditions. Mun GI, Lee SJ, An SM, Kim IK, Boo YC. *Free Radical Biology and Medicine*. 47(3):291-299. 2009

53) *Oenothera laciniata* inhibits lipopolysaccharide induced production of nitric oxide, prostaglandin E_2, and proinflammatory cytokines in RAW264.7 macrophages. Yoon WJ, Ham YM, Yoo BS, Moon JY, Koh JS, Hyun CG. *Journal of Bioscience and Bioengineering*. 107(4), 429–438, 2009

54) Effect of dietary ascorbic acid on growth and non-specific immune responses of tiger puffer, Takifugu rubripes. Eo J, Lee KJ. *Fish & Shellfish Immunology*. 25(5):611-616. Nov. 2008

55) Flavonoids, taxifolin and luteolin

attenuate cellular melanogenesis despite increasing tyrosinase protein levels. An SM, Kim HJ, Kim JE, Boo YC. *Phytotherapy Research*. 22(9):1200-1207. Sep. 2008

56) *p*-Coumaric acid, a constituent of *Sasa quelpaertensis* Nakai, inhibits cellular melanogenesis stimulated by alpha-melanocyte stimulating hormone. An SM, Lee SI, Choi SW, Moon SW, Boo YC. *British Journal of Dermatology*. 159(2):292-299. Aug. 2008

57) Effect of ascorbic acid on bone marrow-derived mesenchymal stem cell proliferation and differentiation. Choi KM, Seo YK, Yoon HH, Song KY, Kwon SY, Lee HS, Park JK. *Journal of Bioscience and Bioengineering*. 105(6):586-94. Jun. 2008

58) A dual mechanism of 4-hydroxy-5-methyl-3[2H]-furanone inhibiting cellular melanogenesis. An SM, Kim HJ, Boo YC. *Journal of Cosmetic Science*. 59(2):117-125. 2008

59) Comparision between ultrasonography (Dermascan C version 3) and transparency profilometry (Skin Visiometer SV600). Lee HK, Seo YK, Baek JH, Koh JS. *Skin Research and Technology.* 14(1):8-12. Feb. 2008

60) Laminar shear stress inhibits lipid peroxidation induced by high glucose plus arachidonic acid in endothelial cells. Mun GI, An SM, Park H, Jo H, Boo YC. *American Journal of Physiology. Heart and Circulatory Physiology.* 295(5):H1966-1973. 2008

61) Improved Apparent Digestibility Coefficient of Protein and Phosphorus by Supplementation of Microbial Phytase in Diets Containing Cottonseed and Soybean Meal for Juvenile Olive flounder (*Paralichthys livaceus*). Pham MA, Lee K-J, Dang TM, Lim S-J, Ko G-Y, Eo J, Oh D-H. *Asia-Australasian Journal of Animal Sciences*. 21:1367-1375. 2008

62) Protective Effect of *Sasa Quelpaertensis* and *p*-Coumaric Acid on Ethanol-induced Hepatotoxicity in Mice. Lee SI, An SM, Mun GI, Lee SJ, Park KM, Park SH, Boo YC. *Journal of the Korean Society for Applied Biological Chemistry.* 51(4):148-154. 2008

63) Evaluating the Nickel Content in Metal Alloys and the Threshold for Nickel Induced Allergic Contact Dermatitis. Kim YY, Kim MY, Park YM, Kim HO, Koh JS, Lee HK. *Journal of Korean Medical Science*. 23(2):315-319. Apr. 2008

64) Evaluation of the effects of a preparation containing asiaticoside on periocular wrinkles of human volunteers. Lee J, Jung E, Lee H, Seo Y, Koh J, Park D. *International Journal of Cosmetic Science.* 30, 167–173. Feb. 2008

65) Effect of mechanical stimulation on the

proliferation of bone marrow-derived human mesenchymal stem cells. Choi K-M, Seo Y-K, Yoon H-H, Song K-Y, Kwon S-Y, Lee H-S, Park J-K. *Biotechnology and Bioprocess Engineering.* 12:601-609. 2007

66) *Gastrodia elata* Blume protects against stress-induced gastric mucosal lesions in mice. An SM, Park CH, Heo JC, Park JY, Woo SU, Seo JH, Lee MS, Cho KJ, Cho HS, Shin HM, Lee SH. *International Journal of Molecular Medicine*. 20(2):209-215. Aug. 2007

67) Effect of essential and nonessential amino acid compositions on *in vitro* behavior of human mesenchymal stem cells. Choi KM, Yoon H-S, Seo Y-K, Song K-Y, Kwon S-Y, Lee H-S, Park YS, Kim Y-J, Park J-K. *Korean Journal of Chemical Engineering*. 24(6):1058-1063. 2007

68) Effect of Camellia japonica oil on human type I procollagen production and skin barrier function. Jung E, Lee J, Baek J, Jung K, Lee J, Huh S, Koh J, Park D. *Journal of ethnophamacology*. 112(1):127-131. May, 2007

69) NF-kappa B 프로모터 활성을 억제하는 식물추출물. 정은선, 현창구, 이지영, 허성란, 고재숙, 이희경, 백지훈, 유병삼, 문지영, 김주호, 박덕훈. *대한화장품학회지* 32(3):135-140. 2007

70) 기계적 자극을 이용한 인체 중간엽 줄기세포로부터 유사－섬유모세포의 분화 유도. 최경민, 서영권, 윤희훈, 권순용, 이화성, 박용순, 손영숙, 송계용, 김영진, 박정극. *Tissue Engineering and Regenerative Medicine.* 3(4):423-431. 2006

71) 피부 주름 평가에 있어서의 피부 거칠기와 진피 치밀도의 상관성 연구. 이희경, 백지훈, 고재숙, 박덕훈, 이종성, 정은선. *대한화장품학회지* 32(2):123-127. June 2006

72) 피부수분 측정법. 백지훈, 고재숙. *대한피부미용학회지.* 4(1):147-154. 2006

73) 제주도 자생식물들에 대한 미백 및 항산화 효능 탐색. 문지영. 김주호. 현진원. 강경아. 고재숙. 서영경. 백지훈. 박덕훈. 이종성. 정은선. 유병삼. *대한화장품학회지* 32(3):161 -171. Sep. 2006

74) 천마추출물의 항산화 및 항암 활성. 허진철, 박자영, 안상미, 이진만, 윤치영, 신흥묵, 권택규, 이상한. *한국식품저장유통학회지.* 13(1):83-87. 2006

75) 내수성 일광제의 일광차단지수 측정방법. 윤태진, 박상영, 고재숙. *대한피부연구학회지* 13(4):105-110. 2006

76) 인체시험을 이용한 화장품의 안전성 평가. 고재숙. *Korean Journal of Cosmetic Dermatology*. 2(1):56-66. 2005

77) 약용곤충추출물 라이브러리를 이용한 항산화 활성의 초고속 검색. 박자영, 허진철, 안상미, 윤은영, 한상미, 황재삼, 강석우, 윤치영, 이상한. *한국식품저장유통학회지*. 12 (5):482-488. 2005

78) Neurotoxic effects of alcohol and acetaldehyde during embryonic development. Lee RD, An SM, Kim SS, Rhee GS, Kwack SJ, Seok JH, Chae SY, Park CH, Choi YW, Kim HS, Cho HY, Lee BM, Park KL. *Journal of Toxicology Environmental Health Part A*. 68(23-24):2147-2162. Dec. 2005

79) 한국여성의 연령별 눈가 주름의 정량분석. 이미영, 김은정, 이희경, 서영경, 이민선, 고재숙. *Journal of the Society of Cosmetic Scientists of Korea*. 30(1):85-91. May 2004

80) Differential gene profiles in developing embryo and fetus after in utero exposure to ethanol. Lee RD, Rhee GS, An SM, Kim SS, Kwack SJ, Seok JH, Chae SY, Park CH, Yoon HJ, Cho DH, Kim HS, Park KL. *Journal of Toxicology Environmental Health Part A*. 67(23-24):2073-2084. Dec. 2004

81) 비침습적 기기 측정법에 의한 한방화장제품이 인체 피부에 미치는 영향 연구. 박성규, 남개원, 이해광, 안성연, 김은주, 이성일, 김연준, 고재숙, 문성준, 장이섭. *The Korean Journal of Herbology*. 19(3):91-97. 2004

82) 伽呩滋陰丹(가미자음단) 에센스가 인체 피부 생리에 미치는 영향. 박성규, 남개원, 이해광, 배지현, 김진한, 김연준, 고재숙, 강승주, 문성준, 장이섭. *Korean Journal of Oriental Physiology and Pathology*. 18(3):729-733. 2004

83) Cigarette smoking associated with premature facial wrinkling: image analysis of facial skin replicas. Koh JS, Kang H, Choi SW, Kim HO. *International Journal of Dermatology*. 41(1):21-27. Jan. 2002

84) 성인형 여드름 환자와 사춘기 여드름 환자의 지질도 및 *Propionibacterium acnes* 수의 비교. 박현정, 최성우, 채경옥, 고재숙, 김형옥, 박연준, *대한피부과학회지*. 38(9):1199-1204. 2000

85) 수종 계면활성제 첩포에 의해 유발된 홍반반응의 육안적 관찰 및 비침습적 측정방법간의 비교분석. 박현정, 최성우, 고재숙, 강현아, 김형옥. *대한피부연구학회지*. 6(3): 153-160. 1999

86) 비침습적 방법을 이용한 정상 한국인의 성별, 부위별 피부특성 측정. 고재숙, 채구석, 김형옥. *대한피부과학회지*. 36(5):855-864. 1998

87) *In vitro* cell recovery method as an alternative to human damaged skin recovery test. An S-S, Nam K-T, Park J-H, Koh J-S. *Korean Journal of Cosmetic Dermatology.* 23(3):97-100. 1997

88) Melanogenesis inhibitor from Paper Mulberry. Jang D-I, Lee B-G, Jeon C-O, Jo N-S, Park J-H, Cho S-Y, Lee H, Koh J-S. *Cosmetics & Toiletries*. 112:59-62. Mar. 1997

89) 임상시험을 통한 3-Amino Propane Phosphoric Acid (3-APPA)의 노화 억제 효과 확인 (제1보). 하재현, 김종일, 강학희, 김영택, 고재숙, 변영훈, 이진선, 이옥섭, *대한화장품학회지*. 22(1):60-69. 1996

90) 광감작성 시험에서의 동물대체 시험법. 이호, 남기택, 고재숙, 박원재. *대한화장품학회지.* 22-1호, 1996

91) *In vitro* alternatives to skin irritation tests. Shin D-S, Kim D-B, Ryu S-R, Lee S-H, Kim P-Y, Koh J-S, Park W-J. *Cosmetics & Toiletries.* 111:61-64. Nov.1996

92) CAMVA(Chorioallantoic Membrane Vascular Assay)를 이용한 계면활성제의 자극 평가에 관한 연구. 고재숙, 안수선, 박종호. *대한화장품학회지.* 21(1):67-83. 1995.

93) 저 자극 마스카라의 인체의 눈에 대한 안전성 연구. 김성미, 김재호, 김종천, 주천기, 고재숙, 이호, 조수연, 박원재, 박희숙. *대한안과학회지*. 36(12):2074-2080. Dec. 1995

94) *In vitro* alternatives to skin irritation test. Shin DS, Kim DB, Ryu SR, Lee SH, Koh JS, Park WS, Kim PY. *응용약물학회지*. 3(3):242-244. 1995

95) *In vivo* 와 *In vitro* 에서의 광독성 시험법의 비교에 대한 연구. 이호, 고재숙, 박원재. *대한화장품학회지.* 19(1):57-76. 1993

96) 자외선 차단 제품의 SPF 측정과 안전성. 김종일, 이병곤, 고재숙. *대한화장품학회지*. 5-26, Sep. 1992

97) 피부 Type 분류를 위한 연령별 피부의 유분, 수분량 및 Microtopography의 관찰. 고재숙. *대한화장품학회*. 15(1):63-70, 1989
